# Role of Su(Hw) zinc finger 10 and interaction with CP190 and Mod(mdg4) proteins in recruiting the Su(Hw) complex to chromatin sites in *Drosophila*

**DOI:** 10.1371/journal.pone.0193497

**Published:** 2018-02-23

**Authors:** Larisa Melnikova, Margarita Kostyuchenko, Alexander Parshikov, Pavel Georgiev, Anton Golovnin

**Affiliations:** 1 Department of Drosophila Molecular Genetics, Institute of Gene Biology, Russian Academy of Sciences, Moscow, Russia; 2 Department of the Control of Genetic Processes, Institute of Gene Biology, Russian Academy of Sciences, Moscow, Russia; Oxford Brookes University, UNITED KINGDOM

## Abstract

Su(Hw) belongs to the class of proteins that organize chromosome architecture and boundaries/insulators between regulatory domains. This protein contains a cluster of 12 zinc finger domains most of which are responsible for binding to three different modules in the consensus site. Su(Hw) forms a complex with CP190 and Mod(mdg4)-67.2 proteins that binds to well-known *Drosophila* insulators. To understand how Su(Hw) performs its activities and binds to specific sites in chromatin, we have examined the previously described *su(Hw)*^*f*^ mutation that disrupts the 10th zinc finger (ZF10) responsible for Su(Hw) binding to the upstream module. The results have shown that Su(Hw)^f^ loses the ability to interact with CP190 in the absence of DNA. In contrast, complete deletion of ZF10 does not prevent the interaction between Su(Hw)^Δ10^ and CP190. Having studied insulator complex formation in different mutant backgrounds, we conclude that both association with CP190 and Mod(mdg4)-67.2 partners and proper organization of DNA binding site are essential for the efficient recruitment of the Su(Hw) complex to chromatin insulators.

## Introduction

Eukaryotic gene expression is regulated by a complex interplay of different regulatory elements. Recently, it has been found that chromosomes in the genomes of higher eukaryotes are organized into a series of discrete topologically associating domains (TADs). [1–5]. In addition, specific interactions between enhancers and promoters, between several promoters, or more complex interactions between multiple enhancers and promoters have been described [6–9]. Mechanisms involved in the formation of chromosome architecture are not yet completely elucidated. However, a model has arisen that a special class of architectural proteins is involved in the organization of specific long-distance interactions between different types of regulatory elements [10–12].

Transcription factors involved in the activity of insulators have been attributed to the category of architectural proteins. Insulators, or chromatin boundaries, are genomic regulatory elements (nucleoprotein complexes) that can block the action of an enhancer on a promoter when interposed between them [12–16]. It was also shown in transgenic lines, that the majority of insulators can support specific distant interactions between enhancers or silencers and promoters [17–22]. Some insulators are able to interpose a barrier between the transcriptionally active chromatin and heterochromatin [21,23–26].

The best studied DNA binding architectural/insulator proteins belong to the large and poorly characterized class of transcription factors with clusters of zinc fingers (ZFs) of C2H2 type [10,27]. The CTCF protein, evolutionary conserved among bilateral metazoans, contains a cluster of 11 ZFs, while the Su(Hw) protein specific to insects contains 12 ZFs also organized into one cluster [28,29]. The C2H2 ZF clusters recognize long DNA binding motif in which each ZF recognizes three nucleotides [30,31]. As follows from limited experimental data, only part of C2H2 domains are usually involved in DNA binding, with the rest being involved in interactions with proteins or RNAs [32,33]. Most of genomic Su(Hw) binding regions have one or two consensus site(s) [34,35], while studies on transgenic lines have shown that only four reiterated binding sites for Su(Hw) can function affectively [36].

The Su(Hw) protein directly interacts with three proteins characterized in detail previously: Mod(mdg4)-67.2, CP190, and E(y)2/Sus1 [37–43]. In addition, several new interactions of the Su(Hw) insulators have recently been described [44–46].

Mod(mdg4)-67.2 and CP190 are BTB/POZ domain proteins that are recruited to chromatin by the Su(Hw) protein [37,40,43]. Mod(mdg4)-67.2 is one of protein isoforms produced by the *mod(mdg4)* gene, also known as *Evar3-93D*, which encodes a large set of proteins containing the BTB domain at the N-terminus and different C-terminal domains [37,40,41]. As shown previously, Mod(mdg4)-67.2 specifically interacts with Su(Hw) through an isoform-specific C-terminal acidic domain. As most of the described BTB domains in higher eukaryotes, the BTB/POZ domain of CP190 forms stable homodimers, while the BTB domain of Mod(mdg4)-67.2 belongs to a specific class of BTB domains that form multimeric complexes [47–51]. Su(Hw) and many other insulator proteins are involved in recruiting of CP190 to the chromatin [43,51–55].

The genome-wide studies have shown that binding sites for the Su(Hw), Mod(mdg4)-67.2, and CP190 proteins could be grouped into two main classes which are characterized by the binding of either Su(Hw) alone (SBS-O) or of all the three proteins (SBS-CM) [35,56,57]. In the interphase nucleus the Su(Hw), CP190, and Mod(mdg4)-67.2 proteins colocalize in speckles, named insulator bodies [43,58–62]. It was speculated [60] that protein complexes are formed in the insulator bodies before binding to the chromatin.

**Fig 1:**
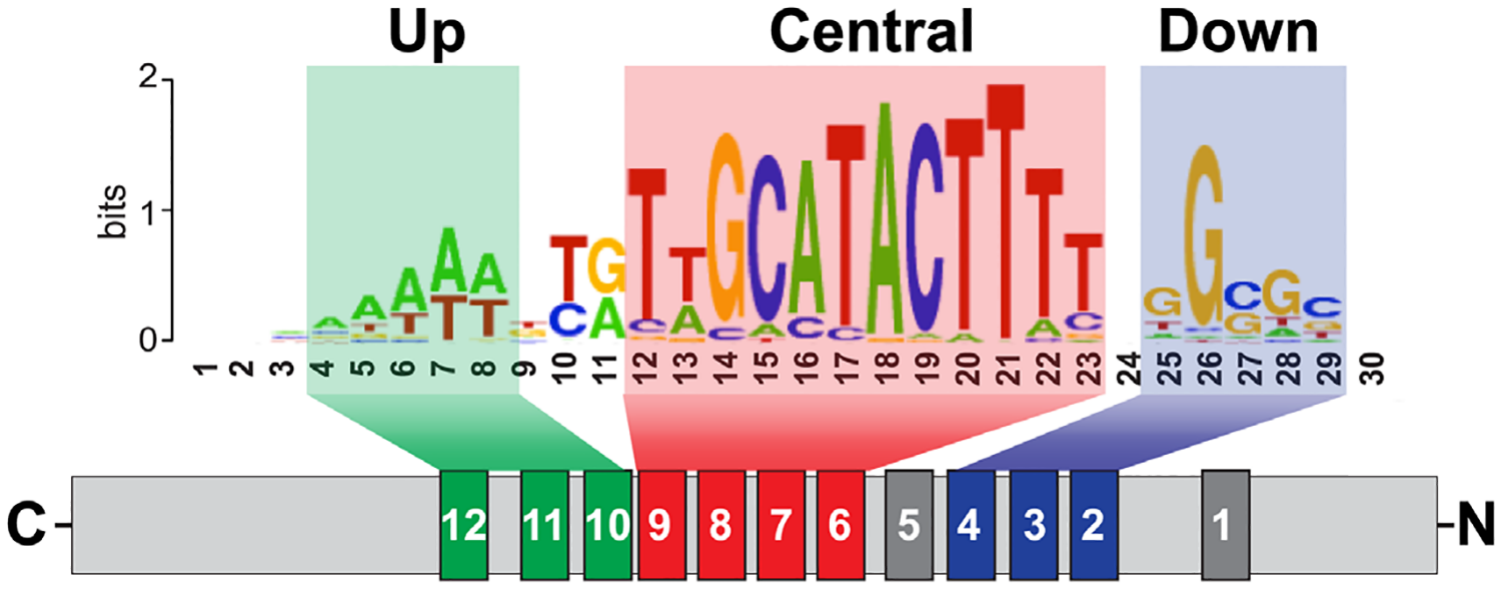
Scheme of Su(Hw) binding with a full consensus binding site, showing which ZFs are involved in recognition of specific cores (from Baxley et al. 2017) [57].

The recent data on the ‘Su(Hw) code’ [56,57] show that Su(Hw) binds to a compound consensus sequence (approximately 26 nucleotides) comprised of three modules. The ZF6–ZF9 cluster binds to the main, central module; the ZF2–ZF4 cluster, to the downstream CG-rich module; and the ZF10–ZF12 cluster, to the upstream AT-rich module (Fig 1). It was shown that the Su(Hw)^f^ protein failed to bind to the *gypsy* insulator consisting from 12 binding sites organized mainly by up and central modules [56,57]. The *su(Hw)*^*f*^ mutation involves the point substitution of the first cysteine in ZF10 with tyrosine [63], which affects the critical Zn-coordinating residue and thereby radically alters the structure of the C2H2 domain. As a result, the Su(Hw^)f^ has inactive the ZF10-ZF12 cluster that is essential for binding to the up AT-rich module in the site. Thus, the Su(Hw)f protein can’t effectively bind to the *gypsy* insulator by using only interaction between the ZF6-ZF9 cluster and the central module in the 12 reiterated sites.

Here we have found that Su(Hw)^f^ loses the ability to interact with CP190 in the absence of DNA. However, deletion of ZF10 (Su(Hw)^Δ10^) restores the CP190–Su(Hw) interaction, suggesting that the point mutation in ZF10 affect conformation of Su(Hw)^f^ leading to loss of interaction with CP190. The Su(Hw)^Δ10^ mutant binds to the *gypsy* insulator better than does Su(Hw)^f^ and partially restores its enhancer-blocking activity but, in contrast to the wild-type Su(Hw) protein, fails to interact with the *gypsy* insulator in the *mod(mdg4)*^*u1*^ mutant background. These results suggest that CP190 and Mod(mdg4)-67.2 are critical for binding of the mutant Su(Hw)^Δ10^ (lacking ZF10) to the *gypsy* insulator consisting mainly of the upstream and central modules. Like the wild-type Su(Hw), the mutant Su(Hw)^f^ protein can efficiently bind to four reiterated binding sites that each consist of three modules. Thus, stable binding of Su(Hw) depends on association with CP190 and Mod(mdg4)-67.2 partners and proper organization of DNA binding site.

## Materials and methods

The constructs for yeast two-hybrid assay, transgenic constructs, and details of experimental and analytical procedures are described in the Supplementary Data (S1 Appendix).

### Germ-line transformation, genetic crosses, and phenotypic analysis

All flies were maintained at 25°C on the standard yeast medium. To obtained transgenic lines carrying a random *P* transposon insertion, the construct together with P25.7wc—a *P* element with defective inverted repeats used as a transposase source [64]–were injected into *y ac w*^*1118*^ preblastoderm embryos. The resulting flies were crossed with *y ac w*^*1118*^ flies, and the transgenic progeny were identified by their eye color. Chromosome localization of various transgene insertions was determined by crossing the transformants with the *y ac w*^*1118*^ balancer stock containing dominant markers, *In(2RL)*,*CyO* for chromosome 2 and *In(3LR)TM3*,*Sb* for chromosome 3. The transformed lines were examined by Southern blot hybridization to check for transposon integrity and copy number.

The lines with excisions of Su(Hw) binding sites were obtained by crossing flies carrying the transposons with the Cre recombinase-expressing line *y*^*1*^, *w*^*1118*^; *CyO*, *P[w*^+^,*cre]/Sco*; +. All excisions were confirmed by PCR analysis.

To test the effects of Su(Hw) protein on *yellow* gene expression, lines containing the *yellow* transposons were crossed into *su(Hw)*^*v*^*/su(Hw)*^*f*^ or *su(Hw)*^*v*^*/su(Hw)*^*e04061*^ mutant background as described previously [38]. *Su(Hw)*^*v*^ is a deletion of the *su(Hw)* gene; *su(Hw)*^*f*^ encodes a protein with a point substitution in ZF10 that retains some ability to bind to DNA; and *su(Hw)*^*e04061*^ is a mutation generated by inserting the PiggyBac element near the start codon [65].

The *yellow* (*y*) phenotype was determined from the level of pigmentation of the abdominal cuticle and wings in 3- to 5-day-old males developing at 25°C. The level of pigmentation (i.e., of *y* expression) was estimated on an arbitrary five-grade scale, with the wild-type expression and the absence of expression assigned scores 5 and 1, respectively. The pigmentation of bristles on the thorax and head was also assessed in 3- to 5-day-old males developing at 25°C. Its variegation was scored on a five-grade scale, with score 1 denoting the loss of pigmentation in all bristles on the thorax and head, score 5 denoting the wild-type pigmentation of all bristles, and intermediate scores being as follows: e-v, extreme variegation (only 1–3 bristles on the thorax and head are pigmented); m-v, moderate variegation (about half of bristles are yellow); and w-v, weak variegation (only 1–3 bristles on thorax and head are yellow). No less than 50 flies from each *y* line were independently scored by two observers.

The *white* (*w*) phenotype was determined from eye pigmentation in adult flies. Wild-type *white* expression determined the bright red eye color (R); in the absence of *white* expression, the eyes were white (W). Intermediate levels of *white* expression (in increasing order) were reflected in the eye color ranging from yellow (Y), through orange (Or), to brown (Br).

To obtain transgenic flies with insertion in 38D, the DNA of reporter constructs was injected into preblastoderm embryos of *y1 M{vas-int*.Dm}ZH-2A w*; M{3xP3-RFP.*attP'}ZH-38D* genotype [66]. The resulting flies were crossed with *y*^*2*^*sc*^*D1*^*w*^*1118*^ *ct*^*6*^ flies, and the progeny carrying the transgene in the 38D region were identified by their eye color. The generation of transgenic lines and construct introduction into the *mod(mdg4)*^*u1*^ or *Su(Hw)*^*v*^*/ Su(Hw)*^*e04061*^ background were performed as described [38].

### Cells lysate preparation and immunoprecipitation experiments

The S2 cells culture was obtained from the stock center of Vavilov Institute of General Genetics, Russian Academy of Sciences (http://vigg.ru/index.php?id=337) [67,68]. The S2 cells cultured as described [69] were transformed using the Effectene Transfection Reagent (Qiagen) as recommended by the manufacturer. To prepare cell lysate, approximately 10^7^ cells were scraped from the plate and washed with two portions of PBS at 4°C, with pelleting at 1000 *g* for 5 min after each wash. The washed pellet was resuspended in 300 μL of lysis buffer (10 mM HEPES, pH 7.9; 450 mM NaCl, 5 mM MgCl_2_, 0.5% NP-40, 1 mM DTT, Protease Inhibitor Cocktail (Roche) and 1U/mL DNase I); incubated on ice for 45 min, with pipetting up and down three to four times to disrupt cell clumps; centrifuged at 15 000 *g*, 4°C, for 15 min; and the supernatant was transferred to a new tube. An aliquot of the supernatant (10% of total volume) was stored to be used as input control #1, and the rest was diluted with four volumes of dilution buffer (10 mM HEPES, pH 7.9; 5 mM MgCl_2_, 1 mM DTT, Protease Inhibitor Cocktail). The diluted lysate was again centrifuged at 15 000 *g*, 4°C, for 15 min, and the supernatant was transferred to a new tube, with an aliquot (10% of total volume) of it being stored as input control #2. The lysate was then supplemented with 40 μL of FLAG Sepharose beads (Sigma) and incubated overnight at 4°C on a rotary shaker. The beads were gently pelleted by centrifugation (700–1000 rpm at 4°C, ~1 min), an aliquot of the supernatant was stored as output control, and the beads were washed with three portions of IP150 buffer (10 mM Tris-HCl, pH 7.5; 150 mM NaCl, 10mM MgCl_2_, 1 mM EDTA, 1 mM EGTA, 0.3 mM DTT, 0.1% NP-40, 10% glycerol), 10 min each, with pelleting between washes. The resulting immunoprecipitate was boiled with 1× Laemmli buffer (25 μL per sample) for 10 min, resolved by SDS-PAGE (20 μL per lane), and immunoblotted with appropriate antibodies (see below).

### Yeast two-hybrid assay

Experiments were performed using plasmids and protocols from Clontech. For growth assays, plasmids were transformed into yeast pJ69-4A cells, which were plated onto media without tryptophan and leucine and grown at 30°C for 3 days. Thereafter, the cells were plated onto selective media without tryptophan, leucine, histidine, and adenine (high stringency conditions) or without tryptophan, leucine, histidine and 5 mM 3-aminotriazole (medium stringency conditions), and their growth was compared after 2–3 days.

### Chromatin preparation and ChIP analysis in pupae

The material (about 150–200 mg of adult male flies, sufficient for four to five independent immunoprecipitations) was homogenized in 5 ml of buffer IP-S+ (10 mM Tris-HCl, pH 7.5; 10 mM NaCl, 10 mM MgCl_2_, 1 mM EDTA, 1 mM EGTA, 1 mM DTT, and 250 mM sucrose with PMSF, leupeptin, and pepstatin A) at 4°C using a Dounce homogenizer with type A pestle. The homogenate was filtered through a BD Falcon filter into a 50-mL tube and pelleted by centrifugation at 4000 *g*, 4°C for 5 min. The supernatant was discarded, and the pellet was washed with three 3-mL portions of buffer IP-S+, with pelleting at 4000 g for 5 min after each wash. The final pellet was resuspended in 0.5 mL of IP-10+ buffer (10 mM Tris-HCl, pH 7.5; 10 mM NaCl, 10 mM MgCl_2_, 1 mM EDTA, 1 mM EGTA, 1mM DTT, 0,1% NP-40, 10% glycerol, and Roche Complete Protease Inhibitor Cocktail) and homogenized at 4°C using a Dounce homogenizer with type B pestle. The homogenate was supplemented with an equal volume of IP-850 buffer (10 mM Tris-HCl, pH 7.5; 850 mM NaCl, 10 mM MgCl_2_, 1 mM EDTA, 1 mM EGTA, 1 mM DTT, 0,1% NP-40, 10% glycerol, and Roche Complete Protease Inhibitor Cocktail), mixed gently, and left on ice for 30–60 min. This was followed by 4 rounds of centrifugation at 14 000 g for 15 min, with the supernatant being each time transferred to a new tube without disturbing the pellet. Immediately before experiments, the lysate was diluted with three volumes of IP-0 buffer (10 mM Tris-HCl, pH 7.5; 10 mM MgCl_2_, 1 mM EDTA, 1 mM EGTA, 1 mM DTT, 0.1% NP-40, 10% glycerol, and Roche Complete Protease Inhibitor Cocktail), centrifuged as above, and transferred to a new tube. To reduce nonspecific background, supernatant was pre-cleared by incubation with protein A or protein G agarose beads at 4°C for 30 min, with constant stirring. Agarose was pelleted by brief centrifugation, and the supernatant was collected for chromatin immunoprecipitation with appropriate antibodies (see below). After overnight incubation at 4°C on a rotary shaker, protein A or protein G agarose beads were added to collect the precipitated complexes, and incubation was continued for 2 h under the same conditions. Agarose was pelleted by centrifugation (700–1000 rpm at 4°C, ~1 min), the supernatant was carefully removed, and the pellet was washed with the following buffers (1 mL each, for 3–5 min on a rotary shaker): Low Salt Wash Buffer (20 mM Tris-HCl, pH 8.0, with 0.1% SDS, 1% Triton X-100, 2 mM EDTA, and 150 mM NaCl), High Salt Wash Buffer (20 mM Tris-HCl, pH 8.0, with 0.1% SDS, 1% Triton X-100, 2 mM EDTA, and 500 mM NaCl), LiCl Wash Buffer (10 mM Tris-HCl, pH 8.0, with 0.25 M LiCl, 1% NP40, 1% deoxycholate, and 1 mM EDTA,), and two portions of TE Buffer. The complex was removed from the agarose by elution with two 250-μL portions of elution buffer (0.1 M NaHCO_3_ with 1% SDS), 15 min each, at room temperature with rotation. The eluates were pooled, supplemented with 20 μL of 5 M NaCl, and heated at 65°C for 4 h to reverse the complex–DNA crosslinks. Then 20 μL of 1 M Tris-HCl (pH 6.5), 10 μL of 0.5 M EDTA, and 2 μL of Proteinase K solution (10 mg/mL) were added, and the mixture was incubated for 1 h at 45°C. DNA was recovered by phenol/chloroform extraction and ethanol precipitation and solubilized in water for PCR. PCR products were amplified from at least two separate immunoprecipitates from three different chromatin preparations. Primer sequences used in PCR for ChIP analysis are shown in the S1 Table.

### Antibodies

Specific antibodies and working dilutions were as follows: monoclonal mouse anti-FLAG (1:300) from Sigma (# F 1804), and polyclonal rat anti-CP190 (1:500), polyclonal rabbit anti-Mod(mdg4)-67.2 (1:500), rabbit anti-Su(Hw) N-terminal domain (1:200), rabbit anti-Chromator (1;300), and rat anti-Skeletor (1:200) raised in our lab and described previously [59,60].

## Results

### The Su(Hw)^f^ protein interacts with Mod(mdg4)-67.2 but not with CP190 *in vitro*

The Su(Hw)^f^ protein in the ovaries fails to bind to 60% of genomic Su(Hw) sites but can still bind to a consensus site *in vitro*, although with a weaker efficiency than the wild-type protein [56]. Since Mod(mdg4)-67.2 and CP190 may be involved in Su(Hw) recruitment to chromatin [35,41,60], we tested the possibility that Su(Hw)^f^ loses the ability to interact with any of these proteins.

**Fig 2:**
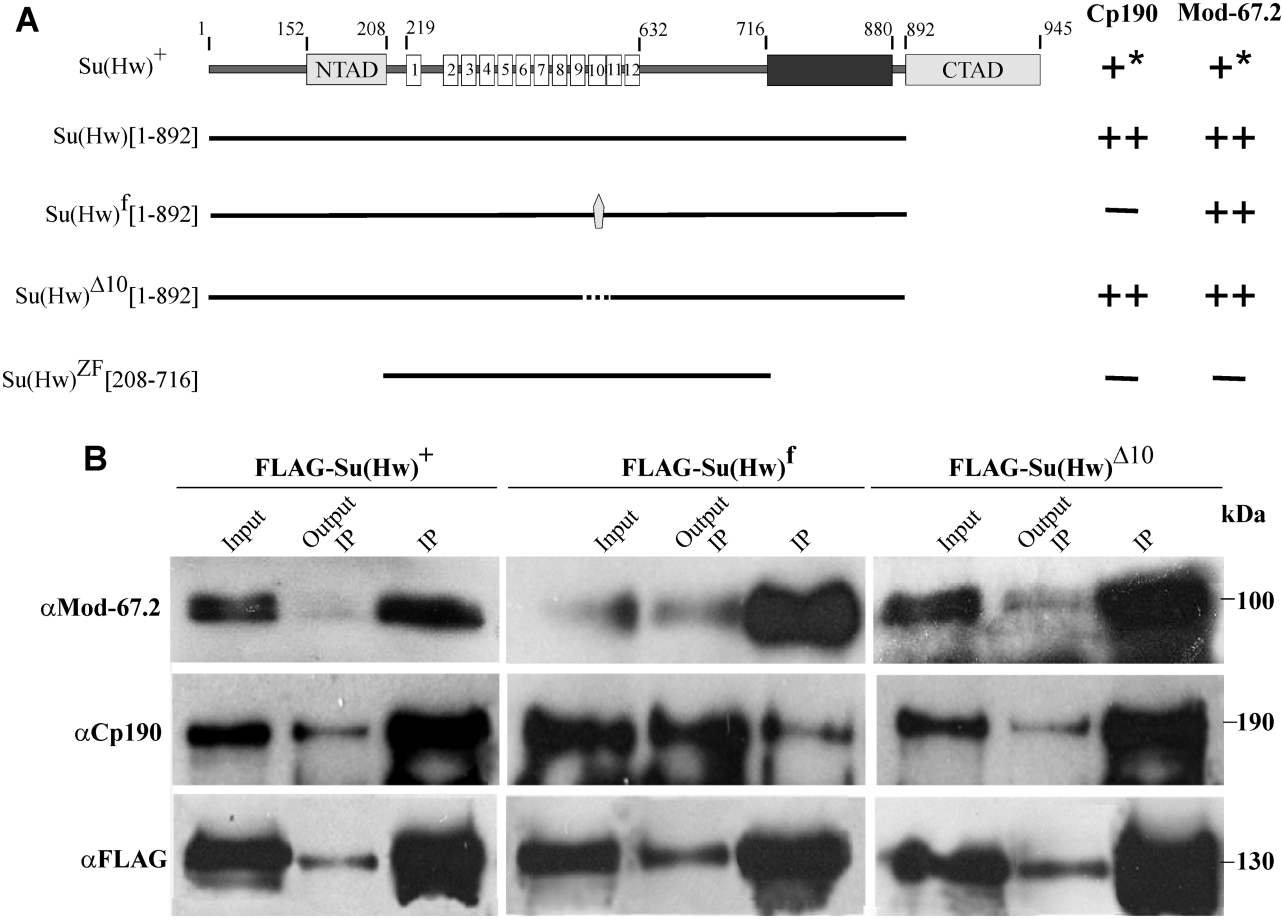
Role of ZF10 in Su(Hw) for interaction between the insulator proteins. A structural schematic of Su(Hw). The borders between the Su(Hw) domains (NTAD, N-terminal acidic domain; CTAD, C-terminal acidic domain; ZF, zinc-finger domain) are indicated by numbers. Gray pentagon indicates H-to-Y substitution in the Su(Hw)^f^ mutant; dotted line, deletion of ZF10 in the Su(Hw)^Δ10^ mutant. The number of plus signs indicates the relative strength of interaction in the Y2H assay (S1 Fig); the minus sign denoted an absence of interactions; an asterisk, a reduction in the Y2H signal due to the repressive effect of Su(Hw) C-terminal domain on the transcription in yeast [72]. All the results were reproduced in three independent experiments. Numbers in brackets refer to the amino acid residues that flank protein regions included in the analysis. (B) Co-immunoprecipitation between different Su(Hw) variants fused to the FLAG epitope and the insulator proteins under normal conditions. The FLAG-Su(Hw)^+^, FLAG-Su(Hw)^f^, and FLAG-Su(Hw)^Δ10^ were expressed in the S2 cells. The immunoprecipitated complexes were washed with buffers containing 150 mM NaCl before loading onto the SDS-PAGE for Western blot analysis. The PVDF membrane was consecutively probed with antibodies against the indicated proteins (CP190 or Mod-67.2) or FLAG epitope. Each column represents a single FLAG-immunoprecipitation experiment with the particular mutant variant. Each lane shows the result of a subsequent hybridization of each immunoprecipitate with different antibodies on the same membrane. "Input" is the input fraction (10% of the lysate used for the immunoprecipitation); "Output IP," the supernatant after the immunoprecipitation; "IP," the immunoprecipitate. The results were obtained in three independent experiments.

The Su(Hw) protein (Fig 2A) contains an N-terminal acidic domain (152 aa– 208 aa), 12 ZF domains (219 aa– 632 aa), and a C-terminal acidic domain (892 aa– 945 aa) [37,63]. It has been shown that Mod(mdg4)-67.2 interacts with the N-terminal domain [70] and the C-terminal domain, including the aa 716 to 880 region [40] (Fig 2A). The BTB domain of CP190 interacts with two adjacent regions in the N-terminal part of Su(Hw) [71].

To test for the interaction between the insulator proteins, we used the yeast two-hybrid assay (Y2H), which is effective for studying complete proteins that are difficult to express in a bacterial *in vitro* system (S1 Fig). The method is based on fusing the GAL4 activation and DNA binding domains to the N- or C-end of the test protein or its part. We compared the interactions of the wild-type and Su(Hw)^f^ mutant with CP190 and Mod(mdg4)-67.2 and found that Su(Hw)^f^ did not interact with Cp190 (Fig 2A), but still interacted with Mod(mdg4)-67.2.

The loss of association between CP190 and Su(Hw) might be explained by a change in Su(Hw) conformation due to a defect in the structure of ZF10. To test this possibility, we used the mutant Su(Hw)^Δ10^ protein in which ZF10 was completely deleted and found that Su(Hw)^Δ10^ interacted with both CP190 and Mod(mdg4)-67.2 like the wild-type protein (Fig 2A). These results suggest that ZF10 is not directly required for the Su(Hw)–CP190 interaction, but structurally defective ZF10 affects the ability of mutant Su(Hw) to interact with CP190.

To confirm the results of Y2H assay, we tested the above interactions in co-IP experiments on S2 cells in which Su(Hw), Su(Hw)^f^, and Su(Hw)^Δ10^ tagged with a triple FLAG epitope were expressed (Fig 2B and S2 Fig). We observed co-immunoprecipitation between FLAG-Su(Hw)^+^ or FLAG-Su(Hw)^Δ10^ and endogenous Mod(mdg4)-67.2 and CP190 proteins. At the same time, FLAG-Su(Hw)^f^ effectively co-precipitated with Mod(mdg4)-67.2 but only weakly co-precipitated with endogenous CP190. Taken together, these results show that structural alteration of ZF10 by point mutation in the Su(Hw)^f^ protein interferes with the ability of the mutant to interact with CP190.

### Normal ZF10 and interaction with CP190 and Mod(mdg4) are essential for binding of Su(Hw) to chromatin

To further compare the role of ZF10 and CP190/Mod(mdg4)-67.2 partners in the binding of Su(Hw) to chromatin sites, we produced transgenic lines expressing either wild-type FLAG-tagged protein (Su(Hw)^+^) or its FLAG-tagged mutant variants (Su(Hw)^f^ and Su(Hw)^Δ10^) under control of the Actin5C (Act5C) promoter. As shown previously, Su(Hw)^f^ in the ovaries and larvae binds only to the “f-retained” sites (such as 62D, 50A, 87E), but not to “f-lost” sites (such as 1A2, 66E, and *gypsy*) [56]. To test the affinity of Su(Hw)^f^ mutant for the “f-lost” sites we also expressed this protein under control of the *ubiquitin-63E* (Ubi) promoter. A *phiC31*-based integration system was used to insert the transgenes at the 38D site. After that, the transgenes were introduced into the *su(Hw)*^−^ background (*su(Hw)*^*v*^*/su(Hw)*^*e04061*^). The expression of the Su(Hw) transgenes was verified by the Western blot analysis with the anti-FLAG antibodies (S3 Fig) The Su(Hw) variants under control of Act5C were expressed at similar levels, while the expression of Su(Hw)^f^ under control of the Ubi promoter was at least two to three times stronger than in that lines.

The interactions between the Su(Hw) variants and Mod(mdg4)-67.2 or CP190 were also assayed by co-IPs from the extract prepared from the 2-day old males of the corresponding transgenic lines (S4 Fig). This experiment confirmed the results obtained in S2 cells: Su(Hw)^f^ showed only weak co-precipitation with endogenous CP190 but normal co-precipitation with Mod(mdg4)-67.2, while Su(Hw)^Δ10^ effectively co-precipitated with both proteins.

**Fig 3:**
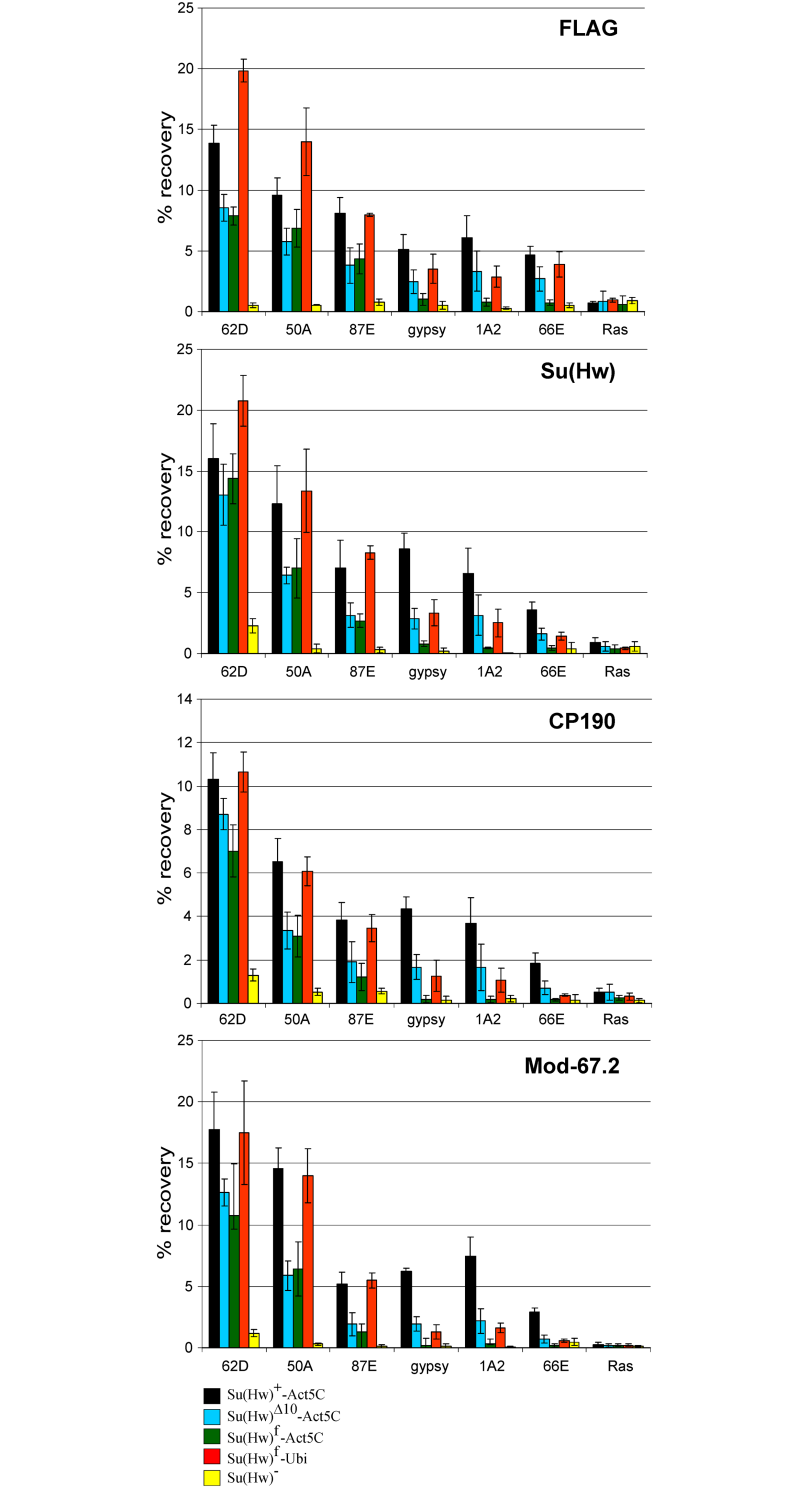
Evaluation of binding of the insulator protein in the *mod(mdg4)*^+^ transgenic lines. Variants of the Su(Hw) protein were expressed in the *y*^*2*^*sc*^*D1*^*ct*^*6*^; *P{Su(Hw)}-38D/P{Su(Hw)}-38D*; *su(Hw)*^*v*^*/su(Hw)*^*e04061*^ lines, where P{Su(Hw)} are Su(Hw)^+^-Act5C –*P{w*^+^;*WAB-Su(Hw)1-945-FLAG}/ P{w*^+^;*WAB-Su(Hw)1-945-FLAG}*; Su(Hw)^Δ10^-Act5C –*P{w*^+^;*WAB-Su(Hw)*^*Δ10*^*-FLAG}/ P{w*^+^;*WAB-Su(Hw)*^*Δ10*^*-FLAG}*; Su(Hw)^f^-Act5C –*P{w*^+^;*WAB-Su(Hw)*^*f*^ *-FLAG}/ P{w*^+^;*WAB-Su(Hw)*^*f*^ *-FLAG}*; Su(Hw)^f^-Ubi–*P{w*^+^;*UbqW-Su(Hw)*^*f*^ *-FLAG}/ P{w*^+^;*UbqW-Su(Hw)*^*f*^ *-FLAG}*. The *y*^*2*^*sc*^*D1*^*ct*^*6*^; *su(Hw)*^*v*^*/su(Hw)*^*e04061*^ line is designated as Su(Hw)^−^. Quantitative PCR (qPCR) was performed at the selected Su(Hw) regions. The *ras64B* coding region (Ras) was used as a negative control. The percent recovery of immunoprecipitated DNA (Y axis) was calculated relative to the amount of input DNA. Error bars indicate standard deviation of three independent biological replicates.

Next, we studied binding of the insulator proteins by ChIP at the previously characterized Su(Hw) sites [73] in pupae of the transgenic lines (Fig 3). As expected, we observed a positive Su(Hw) binding signal at all test sites in the Su(Hw)^+^ transgenic line. In the *su(Hw)*^*−*^ pupae, we still observed residual Su(Hw) binding to insulator site 62D, which was correlated with Mod(mdg4)-67.2 binding to the same site. Residual binding of CP190 was observed at 62D, 50A and 87E sites. These results are in agreement with data on the critical role of Su(Hw) in recruiting of the Mod(mdg4)-67.2 isoform [40], while CP190 is known to be recruited to chromatin by many DNA-binding proteins [35,54].

In the transgenic line expressing Su(Hw)^f^ under control of the Act5C promoter, this protein was found to bind to the f-retained 62D, 50A, and 87E sites, similar to wild-type Su(Hw), but not to the f-lost sites 1A2, 66E, and *gypsy* (Fig 3). We also observed that the Mod(mdg4)-67.2 and CP190 proteins were recruited to the 62D, 50A, 87E sites but not to the 1A2, 66E and *gypsy* sites, suggesting that Su(Hw)^f^ was able to recruit the CP190 and Mod(mdg4)-67.2 proteins to chromatin. Thus, Su(Hw)^f^ failed to bind the *gypsy* and 1A2 sites in female gonads [56], larvae [73] and pupae (this study).

In contrast, Su(Hw)^Δ10^ bound not only to the 62D, 50A, 87E sites but also (at a medium level) to the 1A2, 66E, and *gypsy* insulators. The C190 and Mod(mdg4)-67.2 proteins were also recruited to the 1A2, 66E, and *gypsy* sites. These results showed that Su(Hw)^Δ10^, compared to Su(Hw)^f^, can better bind to the f-lost sites. However, when Su(Hw)^f^ was expressed at a higher level (under control of the Ubi promoter), we observed not only better binding of Su(Hw)^f^ to the 62D, 50A, 87E sites, but also moderate binding of the mutant protein to the 1A2, 66E and *gypsy* sites Thus, the increasing expression of Su(Hw)^f^ provides for its binding to the f-lost sites (Fig 3).

**Fig 4:**
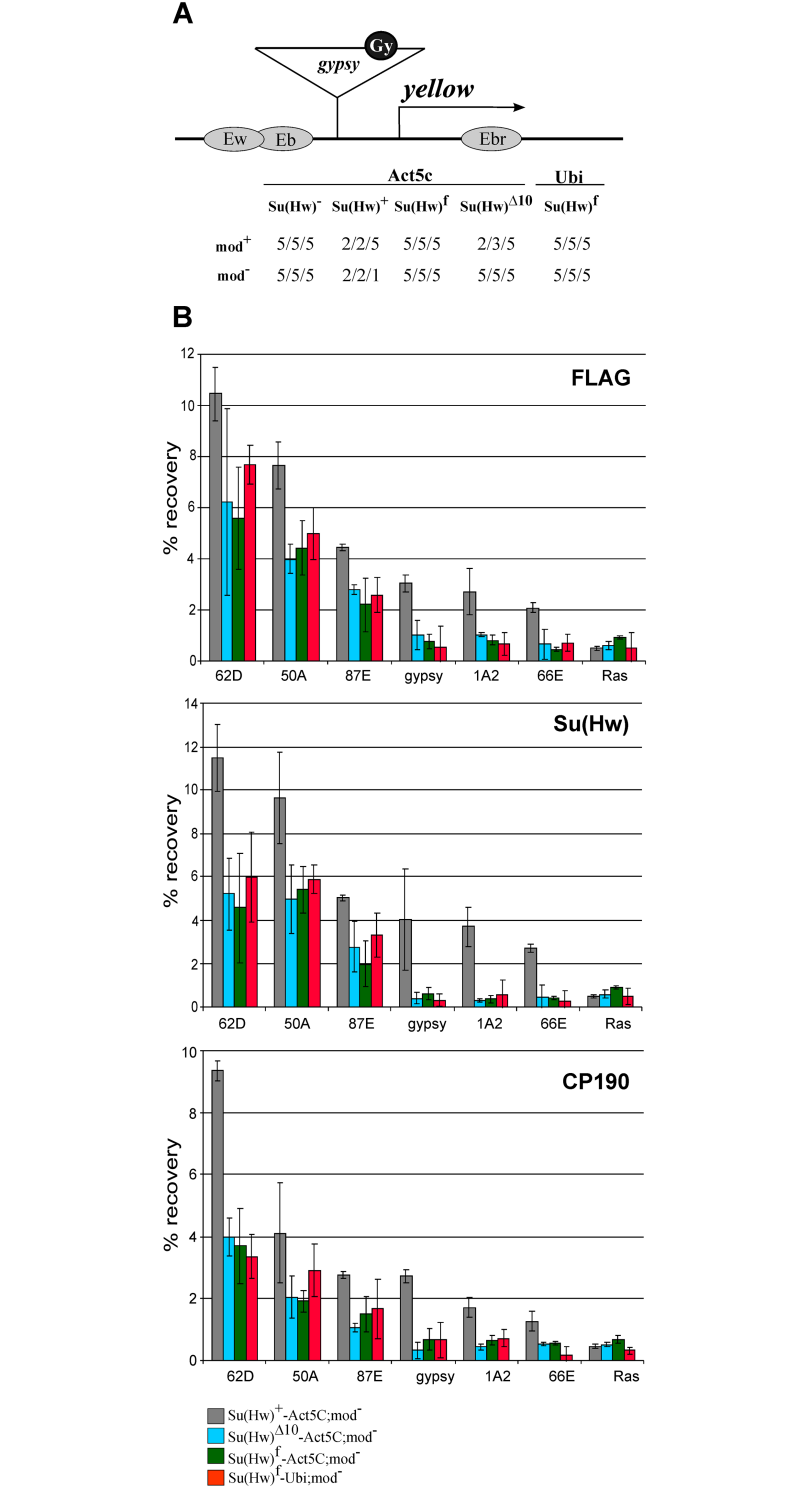
Effects of the su(Hw) mutations on the activity of *gypsy* insulator in the *mod(mdg4)*^*u1*^ background. (A) Effects of the *mod(mdg4)*^*u1*^ mutation on *yellow* expression in transgenic lines. A schematic of the *y*^*2*^ allele (drawn not to scale): the *yellow* wing (Ew) and body (Eb) enhancers are shown as partially overlapping gray ovals; the bristle enhancer (Ebr), as a gray oval in the *yellow* intron; the transcription start site is indicated by an arrowhead. The *gypsy* insertion is shown as a triangle with the black circle (Gy) marking Su(Hw) binding sites. Analysis of the transgenic lines was performed in the *y*^*2*^*sc*^*D1*^*ct*^*6*^; *mod(mdg4)*^+^*/mod(mdg4)*^+^ background (mod^+^) or in the *y*^*2*^*sc*^*D1*^*ct*^*6*^; *mod(mdg4)*^*u1*^*/mod(mdg4)*^*u1*^ background (mod^−^). Numbers in the "mod^+^" and "mod^Ȣ^" lines indicate a relative level of *yellow* expression in the body cuticle/wing blades/bristles, which ranged from 2 (pigmentation as in the *y*^*2*^ allele) to 5 (pigmentation as in the wild-type flies). (B) ChIP-qPCR analysis of Su(Hw), Mod-67.2, and CP190 binding at the mid-pupal stage in the transgenic lines expressing different variants of Su(Hw) in the *y*^*2*^*sc*^*D1*^*ct*^*6*^; *P{Su(Hw)}-38D/P{Su(Hw)}-38D*; *su(Hw)*^*v*^ *mod(mdg4)*^*u1*^*/ su(Hw)*^*e04061*^ *mod(mdg4)*^*u1*^ lines (mod^−^ in designations of the lines). The abbreviations of transgenes P{Su(Hw)} are as in Fig 3. Quantitative PCR (qPCR) was performed on the intergenic regions bound by Su(Hw). PCR products were amplified from two separate immunoprecipitates of three different chromatin preparations. The *ras64B* coding region (Ras) was used as a control devoid of Su(Hw) binding sites. The recovery percentage of immunoprecipitated DNA (Y axis) was calculated relative to the amount of input DNA. Error bars indicate standard deviation of three independent biological replicates.

These results correlated with phenotypes of the *y*^*2*^ allele in different Su(Hw) backgrounds (Fig 4A). In the *y*^*2*^ mutation (Fig 4A), *gypsy* is inserted between the *yellow* promoter and the enhancers controlling expression in the wings and body cuticle [74]. The Su(Hw) insulator blocks activity of the enhancers that results in a weak *yellow* expression in wing blades and body cuticle (*y*^*2*^ phenotype) [74,75]. In flies bearing a combination of Su(Hw)^f^ and *y*^*2*^ *yellow* expression was completely restored, like in *y*^*2*^; *su(Hw)*^−^ flies. In contrast, the expression of Su(Hw)^Δ10^ supported the enhancer-blocking activity of the *gypsy* insulator in the *y*^*2*^ allele, as in the case of Su(Hw)^+^. Thus, Su(Hw)^Δ10^ binds to the *gypsy* sequences and almost completely restores its enhancer-blocking activity. On the other hand, the overexpression of Su(Hw)^f^ failed to restore even partial blocking of the *yellow* enhancers by the *gypsy* insulator in the *y*^*2*^ allele.

We also tested the effect of different Su(Hw) variants on the activity of the *gypsy* insulator in *cut* and *scute* loci (S5 Fig). In the *ct*^*6*^ allele, *gypsy* is located between the *cut* promoter and the wing margin enhancer separated by 85 kb [37]. The mutant phenotype in the *ct*^*6*^ allele is a consequence of complete blocking of the margin enhancer by the insulator. In *sc*^*D1*^ allele, downstream insertion of *gypsy* inhibits *scute* expression in several specific areas of the epithelium and, in particular, blocks the formation of several types of bristles [76]. All Su(Hw) mutant variants expressed in the *su(Hw)*^−^ background (*su(Hw)*^*v*^*/su(Hw)*^*e04061*^) failed to support the ability of the *gypsy* insulator to block *cut* or *scute* enhancer in the *ct*^*6*^ and *sc*^*D1*^ alleles, showing that Su(Hw)^Δ10^, like Su(Hw)^f^, does not support the enhancer-blocking activity of the *gypsy* insulator in tissues where the *cut* or *scute* genes are required.

Taken together, these results indicated that the CP190–Su(Hw) interaction has an effect on binding of the Su(Hw) complex to the chromatin. The Su(Hw)–Mod(mdg4)-67.2 interaction was also suggested to have a role in this process [60]. To test the role of Mod(mdg4)-67.2 in the binding of Su(Hw)^Δ10^ and Su(Hw)^f^, the Su(Hw) variants were expressed in the *su(Hw)*^−^(*su(Hw)*^*v*^/*su(Hw)*^*e04061*^); *mod(mdg4)*^*u1*^ background (Fig 4B). The result showed that inactivation of Mod(mdg4)-67.2 only slightly affected the binding of Su(Hw)^+^ to the chromatin but almost completely prevented Su(Hw)^Δ10^ and Su(Hw)^f^ binding to the f-lost sites and strongly reduced their binding to the f-retained sites.

As shown previously, the *mod*(*mdg4*)^*u1*^ mutation leads to a direct repression of *yellow* in *y*^*2*^ flies that results in the nonpigmented bristles and a variegated pigmentation in the abdominal segments. We found that *mod*(*mdg4*)^*u1*^ in combination with Su(Hw)^Δ10^ transgene almost completely suppressed the *y*^*2*^ mutant phenotype, indicating that Su(Hw)^Δ10^ failed to bind the *gypsy* sequence in the absence of the Mod(mdg4)-67.2 protein (Fig 4B).

### Comparing the functional activity of the *gypsy* insulator and four Su(Hw) binding sites

The Su(Hw) protein binds to the sites integrated in the regulatory regions that are also bound by unknown transcription factors, which may facilitate the binding of Su(Hw)^f^ to DNA. To find out whether the presence of an appropriate DNA binding motif is sufficient for recruiting the Su(Hw)^f^ protein, we used a DNA fragment with four reiterated Su(Hw) motifs (S^×4^) corresponding to site 3 in the *gypsy* insulator (S6 Fig). This fragment contained all three modules corresponding to the consensus site.

**Fig 5:**
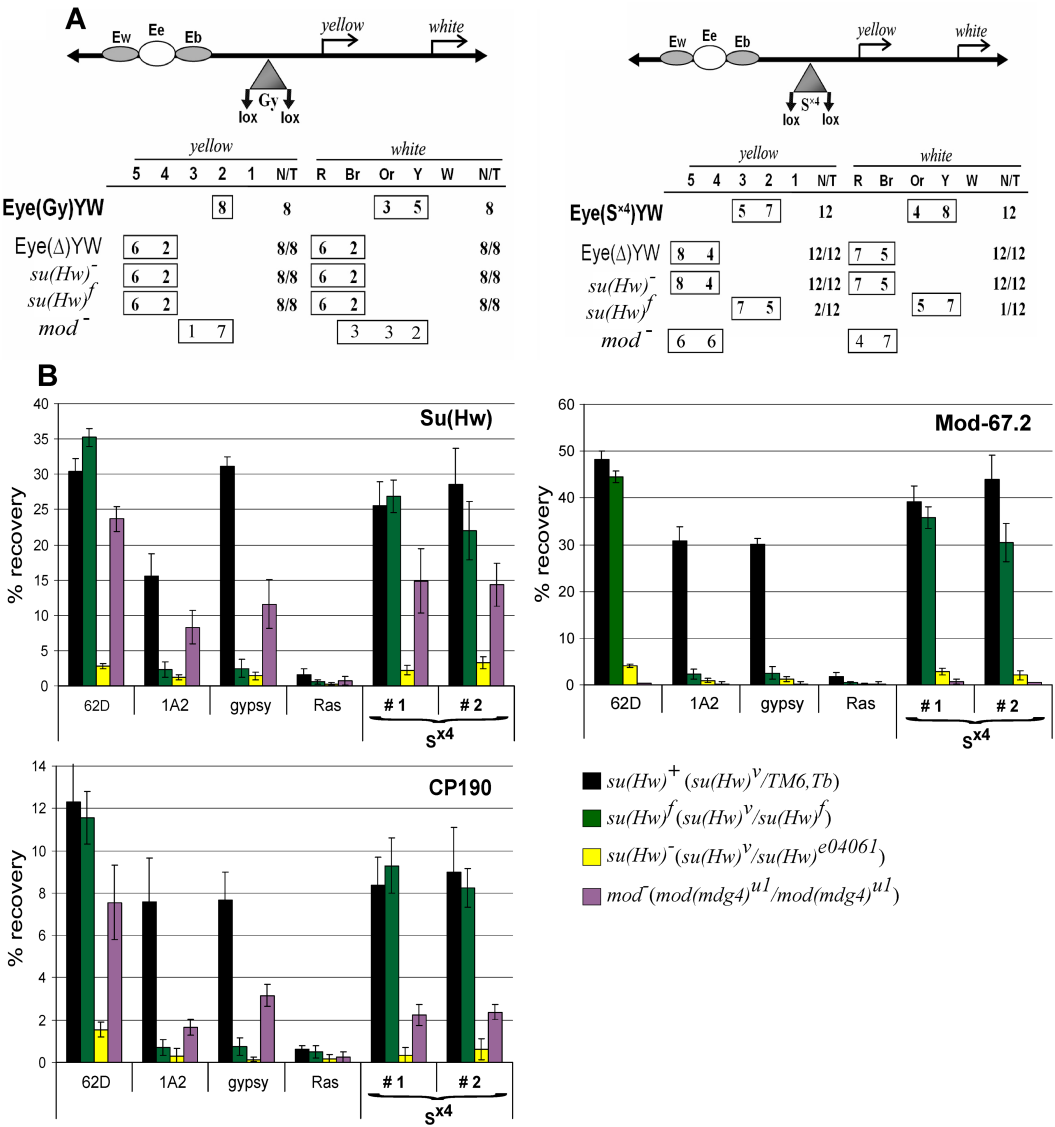
Comparison of the enhancer-blocking activity of the *gypsy* (Gy) and S^x4^ insulators. (A) Effects of the *su(Hw)* and *mod(mdg4)* mutations on *yellow* and *white* expression in transgenic lines. In the scheme of the construct (drawn not to scale), the *yellow* wing (Ew) and body (Eb) enhancers are shown as shaded ovals; the eye enhancer (Ee) inserted between them, as a white oval; the *yellow* (Y) and *white* (W) genes, as arrows indicating the direction of transcription; and the *gypsy* and S^×4^ insulators, as shaded triangles. Downward arrows indicate *loxP* target sites for the Cre recombinase; the same sites in the construct names are denoted by parentheses. The “*yellow*” column shows the numbers of transgenic lines with the *yellow* pigmentation level in the abdominal cuticle (reflecting the activity of the body enhancer); in most of the lines, the pigmentation levels in wing blades (reflecting the activity of the wing enhancer) closely correlated with these scores. The level of pigmentation (i.e., of *y* expression) was estimated on an arbitrary five-grade scale, with wild-type expression and the absence of expression assigned scores of 5 and 1, respectively. Wild-type *white* expression determined the bright red eye color (R); in the absence of *white* expression, the eyes were white (W). Intermediate levels of pigmentation, with the eye color ranging from pale yellow to yellow (Y) from dark yellow to orange (Or), from dark orange to brownish red (Br) reflect the increasing levels of *white* expression. In the N/T ratio, N is the number of lines in which flies acquired a new *y* phenotype upon deletion (Δ) of the specified DNA fragment, or on the mutant background, and T is the total number of lines examined for each particular construct. (B) ChIP-qPCR analysis of insulator proteins binding to the *gypsy* or S^×4^ insulator in transgenic lines. The Eye(S^×4^)YW lines included in the analysis are designated # 1 and # 2. The *ras64B* coding region (Ras) was used as a control devoid of Su(Hw) binding sites. The percent recovery of immunoprecipitated DNA (Y axis) was calculated relative to the amount of input DNA. Error bars indicate standard deviation of three independent biological replicates. The abbreviations of mutant backgrounds: *su(Hw)*^+^—*su(Hw)*^*v*^/*TM6,Tb*; *su(Hw)*^‒^—*su(Hw)*^*v*^/*su(Hw)*^*e04061*^; *su(Hw)*^*f*^–*su(Hw)*^*v*^/*su(Hw)*^*f*^; *mod*^-^–*mod(mdg4)*^*u1*^/*mod(mdg4)*^*u1*^.

We compared the Su(Hw)^+^ and Su(Hw)^f^ proteins for binding to the S^×4^ element and the ability to mediate the enhancer-blocking and barrier activity of the Su(Hw) sites. As reporters, we used the *yellow* gene and the *white* gene that was inserted on the 3’ side of *yellow* in tail-to-head orientation. The *white* gene accounts for eye pigmentation and is regulated by the eye-specific enhancer [77]. To test the enhancer-blocking activity, constructs were made (Fig 5A), in which the eye enhancer was inserted between the *yellow* wing and body enhancers, and the S^×4^ fragment flanked by *loxP* sites was inserted between the enhancers and promoters at –893 relative to the *yellow* transcription start site. As a control, we used the previously characterized transgenic line carrying the construct with the same configuration of the regulatory elements and the *gypsy* insulator flanked by the *loxP* sites (Fig 5A). We obtained 12 transgenic lines, each carrying one copy of the construct with the S^×4^ sites. The S^×4^ fragment effectively blocked the *yellow* and *white* enhancers, with deletion of S^×4^ restoring the expression of the reporter genes. These results showed that the S^×4^ sites blocked the enhancers at a comparable level with the *gypsy* insulator (Fig 5A and S7 Fig). In the *su(Hw)*^*v*^*/ su(Hw)*^*f*^ background, the Su(Hw)^f^ protein completely blocked the enhancers in transgenic lines carrying the S^×4^ but not in transgenic lines carrying the *gypsy* insulator (Fig 5A). As expected, both test elements lost their enhancer-blocking activity in the *su(Hw)*^−^(*su(Hw)*^*v*^*/ su(Hw)*^*e041064*^) background. Taken together, these results showed that S^×4^ sites were fully active in the transgenic lines expressing the mutant Su(Hw)^f^ protein.

In two independent S^×4^ transgenic lines, binding of the Su(Hw), Mod(mdg4)-67.2, and CP190 proteins was tested by means of ChIP, with the 62D and 1A2 sites being used as an internal control. The binding of these proteins was also tested in the control transgenic line carrying the *gypsy* insulator. Chromatin was isolated from pupae of transgenic lines with either *su(Hw)*^*v*^*/TM6*,*Tb*, *su(Hw)*^*v*^*/su(Hw)*^*e041064*^ or *su(Hw)*^*v*^*/su(Hw)*^*f*^ background (Fig 5B). The results showed that all three proteins—Su(Hw), CP190 and Mod(mdg4)-67.2– bound to the S^×4^ sites in the two selected transgenic lines and to the *gypsy* insulator in the *su(Hw)*^*v*^*/TM6*,*Tb* background, while the Su(Hw)^f^ protein effectively bound to the S^×4^ sites but not to the *gypsy* insulator. Thus, Su(Hw)^f^ proved to bind to the reiterated four sites containing all three modules (S5 Fig). Importantly, CP190 bound to the S^×4^ sites with the same efficiency in both *su(Hw)*^+^ and *su(Hw)*^*f*^ backgrounds. These results confirm that CP190 is able to interact with the Su(Hw) sites after its binding to DNA.

According to the ‘Su(Hw) code’ model, the DNA binding site determines which of the ZF domains are not involved in DNA binding and may therefore be available for association with additional proteins such as E(y)2/Sus1 [42,56,57]. To test whether *gypsy* and S^×4^ display different properties, we compared their activity in transgenic lines crossed into the *mod(mdg4)*^*u1*^ background (Fig 5A). The inactivation of Mod(mdg4)-67.2 in the *mod(mdg4)*^*u1*^ mutation had little effect on the activity of the *gypsy* insulator, which was manifested in the partial release of the eye enhancer, while the *yellow* enhancers remained inactive. In contrast, the *mod(mdg4)*^*u1*^ mutation led to almost complete inactivation of the enhancer-blocking activity of the S^×4^ insulator. Interestingly, inactivation of *mod(mdg4)*^*u1*^ only moderately affected the binding of Su(Hw) and CP190 to both *gypsy* and S^×4^ insulators (Fig 5B). Thus, the Su(Hw) and CP190 proteins still bound to the sites, but their ability to block enhancers was lost only in the case of the S^×4^ sites.

**Fig 6:**
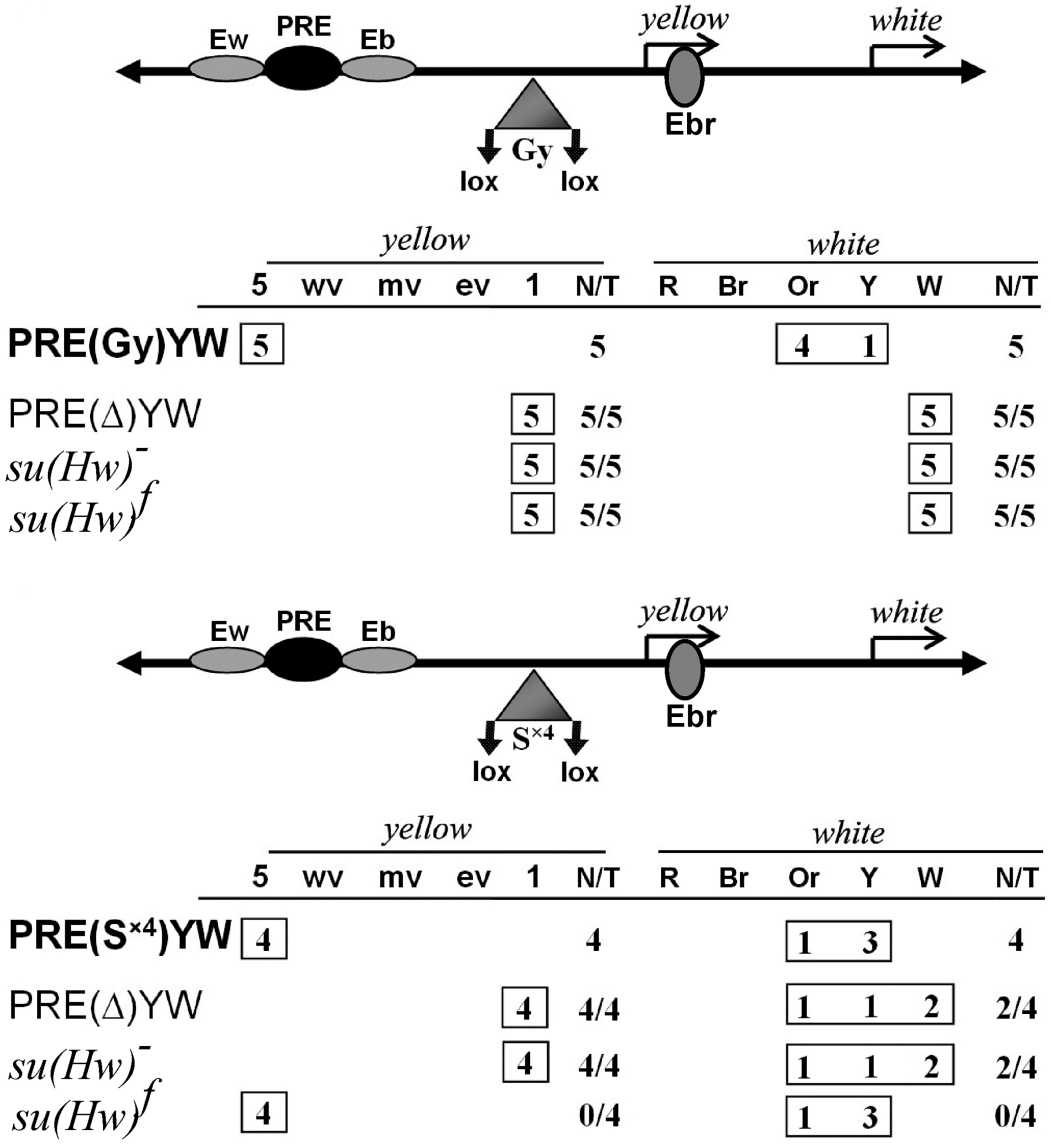
Comparison of the antisilencing activity of *gypsy* and S^×4^ insulators. The 660-bp PRE is shown as a black oval; the bristle enhancer (Ebr), as a gray oval in the intron of the *yellow* gene. The “*yellow*” column shows the numbers of transgenic lines with the *yellow* pigmentation level in bristles. The degree of variegation in bristles of the thorax and head: 1, loss of pigmentation in all bristles at thorax and head; e-v, extreme variegation (only 1–3 bristles on thorax and head are pigmented); m-v, moderate variegation (about half of bristles are yellow); w-v, weak variegation (only 1–3 bristles on thorax and head are yellow); 5, pigmentation of all bristles as in wild-type flies. Other designations are as in Fig 5.

In addition to blocking the enhancers, one copy of the *gypsy* insulator can also counteract the repressive effect of the Polycomb Response Element (PRE) [17,78,79]. PREs are bound by proteins of the Polycomb group (PcG), inducing the silencing of both endogenous target genes and reporter genes [80]. As shown previously, E(y)2/Sus1 is recruited to the Su(Hw) insulators and is required for blocking of PRE-mediated repression by the *gypsy* insulator [42]. Since ZFs 7–12 were mapped to interact with E(y)2/Sus1, we ask whether S^×4^ functions differently from the *gypsy* insulator in protecting reporter expression from PRE-mediated repression. In the constructs (Fig 6), the 660-bp core PRE from the *bxd* region of homeotic gene *Ultrabithorax* [79] was inserted between the wing and body enhancers. The *loxP*-flanked S^×4^ fragment was inserted at –893 between the *yellow* promoter and PRE. Only those transgenic lines were included in this experiment in which the PRE repressed the *yellow* and *white* genes after deletion of the insulators. As a result, we found that S^×4^ effectively blocked PRE-mediated silencing of the *yellow* and *white* expression (Fig 6). Crossing into the *su(Hw)*^−^ background resulted in the silencing of *yellow* and *white* expression. Once again, S^×4^ could protect *yellow* and *white* expression from PRE-mediated silencing in the *su(Hw)*^*v*^*/su(Hw)*^*f*^ background. Thus, inactivation of ZF10 in the Su(Hw)^f^ protein does not affect the ability to block PRE-mediated silencing.

## Discussion

Here we have found that the Su(Hw)^f^ protein cannot interact with CP190 in the absence of DNA. It is likely that structural alteration of ZF10 leads to a change in the conformation of the Su(Hw) protein that prevents the interaction of its N-terminal domain with CP190. The proper conformation of Su(Hw) is restored after binding to DNA, which allows the interaction between its N-terminal domain and the BTB domain of CP190. This finding makes it possible to estimate the role of the Su(Hw)–CP190 interaction in recruiting the insulator complex to chromatin.

**Fig 7:**
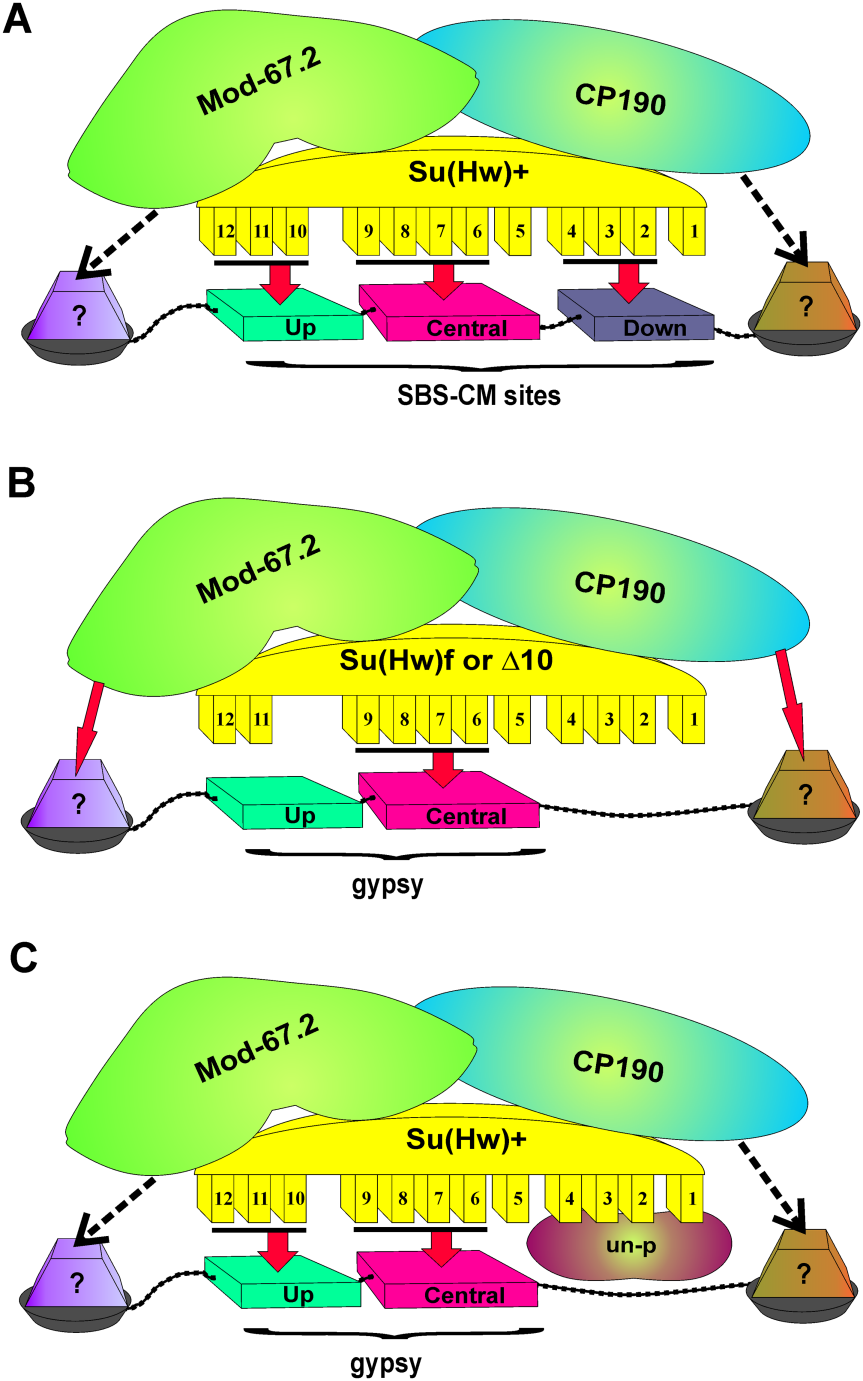
A model explaining differences in the Su(Hw) complex recruitment to the consensus sites and to sites in the *gypsy* insulator. (A) The Su(Hw) complex is recruited to consensus site(s) consisting of three modules. Most of ZFs are involved in stable interaction with the consensus site. The interactions of C190 and Mod(mdg4) with neighboring proteins (dotted arrows) are not critical for recruiting the insulator complex. (B) The insulator complex carrying Su(Hw) with mutated ZF10 is recruited to the sites in the *gypsy* insulator. The binding of the Su(Hw) complex to DNA is unstable and strongly depends on CP190 and Mod(mdg4)-67.2 (thick arrows) that may stabilize interaction with surrounding proteins. (C) The insulator complex with the Su(Hw)^+^ protein is recruited to the *gypsy* sites. ZFs 1–4 are not involved in binding to these sites and may recruit unknown proteins/complex (un-p) that improve the enhancer-blocking activity of the Su(Hw) complex in the absence of the Mod(mdg4)-67.2 protein.

In the *Drosophila* interphase cell nucleus, the Su(Hw), Mod(mdg4)-67.2, and CP190 proteins colocalize in speckles (insulator bodies) that might function as a depot of proteins involved in the regulation of transcription and insulation [60,61,81,82]. The insulator bodies could facilitate a formation of complexes between Su(Hw)–Mod(mdg4)-67.2–CP190 and other transcription factors. It appears that Mod(mdg4)-67.2 and CP190 interact with neighboring DNA binding (architectural) proteins and in this way facilitate the recruitment of ‘mature’ Su(Hw) insulator complexes to the corresponding chromatin sites. The efficiency of Su(Hw) complex recruiting depends on the presence of essential modules in the consensus site, the number of such sites, and the interaction with neighboring partners (Fig 7A). According to the ‘Su(Hw) code’ [56,57], ZF10 and ZF4 of the Su(Hw) protein are required for interaction with the upstream AT-reach module and the downstream CG-reach module of the consensus binding site (Fig 1). The Su(Hw)^f^ protein looses the ability to recognize the upstream module at DNA and at the same cannot interact with CP190 before recruitment to chromatin (Fig 7B). For this reason, only overexpressed Su(Hw)^f^ can weakly bind to the 12 sites in the *gypsy* insulator. It seems likely (although not confirmed) that Su(Hw)^f^ binds only to the third site in the *gypsy* insulator that contains all three modules. This may explain the inability of overexpressed Su(Hw)^f^ to block the *yellow* enhancers, in contrast to Su(Hw)^Δ10^ that interacts with CP190. The relatively weak binding of Su(Hw)^Δ10^ to the *gypsy* insulator is indicative of the main contribution of ZF10 to the Su(Hw) complex recruitment to the *gypsy* sites. Moreover, inactivation of Mod(mdg4)-67.2 also completely blocks the recruitment of Su(Hw)^Δ10^ to the *gypsy* insulator and other “f-lost” sites. Thus, the recruitment of the mutant Su(Hw) protein incapable of recognizing the upstream module in the consensus site is completely dependent on Mod(mdg4)-67.2 and CP190 partners.

The Su(Hw) code model [57] predicts that the consensus site determine which of Su(Hw) ZF domains are not involved in DNA binding and, hence, are free to interact with other proteins and/or complexes (Fig 7C). For example, the ZF domains of the Su(Hw) and dCTCF proteins are required for recruitment of E(y)2/Sus1, which is involved in blocking of PRE-mediated silencing [11,42]. The interaction of E(y)2/Sus1 with Su(Hw) was previously mapped to the region of ZFs 7–12 [42]. Here we found that ZF10 is not required for blocking of PRE by the Su(Hw) sites. Thus, it appears that E(y)2/Sus1 interacts with ZF11 and/or ZF12, which are not highly required for DNA binding [57].

Interestingly, the *gypsy* insulator (12 sites with different modules structure) and four Su(Hw) sites (S^×4^) with three modules show the same efficiency in blocking the *yellow* enhancers in transgenic lines. Inactivation of Mod(mdg4)-67.2 did not significantly change the enhancer-blocking activity of the *gypsy* insulator. At the same time, Mod(mdg4)-67.2 is critical for the insulator activity of the S^×4^ sites. Since Mod(mdg4)-67.2 inactivation only weakly affected binding of the Su(Hw) and CP190 proteins to both *gypsy* and S^×4^ sites, this difference in insulation may be explained by the existence of an unknown protein/complex involved in enhancer blocking that is recruited to the *gypsy* but not to the S^×4^ site. For example, this unknown protein/complex can supposedly interact with ZF4, which is essential for binding to the S^×4^ sites but not to *gypsy*. Further study is required for testing the relevance of the code model.

## Conclusions

The mechanisms regulating the chromatin binding of architectural proteins, such as Su(Hw), are not yet well understood. Our results support the model that the recruitment of the Su(Hw) complex depends on many protein–protein and DNA–protein interactions at the target chromatin sites.

## Supporting information

S1 Appendix Materials and methods. (DOCX)

S1 Table Primer sequences used in PCR for ChIP analysis. (DOC)

S1 Fig Test for two-hybrid interaction strength. (1)—no interaction « − », (2)—« + », (3)—« ++ ».
(TIF)

S2 Fig Co-immunoprecipitation between Su(Hw) variants fused to the FLAG epitope and the Skeletor protein. The FLAG-Su(Hw)^+^, FLAG-Su(Hw)^f^, and FLAG-Su(Hw)^Δ10^ were expressed in the S2 cells. The immunoprecipitated complexes were washed with buffers containing 150 mM NaCl before loading onto the SDS-PAGE for Western blot analysis. The PVDF membrane was probed with antibodies against the Skeletor protein. Each panel represents a single FLAG immunoprecipitation experiment with the particular Su(Hw) variant. Su(Hw) variants do not interact with the Skeletor protein. The bottom panel shows the result of immunoprecipitation between Chromator and Skeletor proteins that interact with each other [83]. All results were reproduced in two independent experiments.
(TIF)

S3 Fig Western blot analysis for the expression of Su(Hw) protein and its derivatives *in vivo*. Su(Hw) isolated from adult flies as described (Gdula et al., 1997) [84] was resolved by electrophoresis in 7.5% SDS-PAAG, electroblotted onto a PVDF membrane, and probed with antibodies against Su(Hw) or FLAG epitope (designated αSu(Hw) and αFLAG, respectively). Anti-tubulin staining (αTub) was used as loading control. The name of alleles included in analysis are indicated above the figure. Expression analysis of transgenic lines was performed on the *y*^*2*^*sc*^*D1*^*ct*^*6*^; *su(Hw)*^*v*^*/ su(Hw)*^*e04061*^ background.
(TIF)

S4 Fig Results of co-immunoprecipitation between the Su(Hw) protein and its derivatives fused to FLAG epitope and the Mod(mdg4)-67.2 or CP190 protein. The immunoprecipitated complexes were washed with 150 mM NaCl-containing buffers before loading onto SDS-PAGE for Western blot analysis. The PVDF membrane was consecutively probed with antibodies against the indicated proteins (CP190 or Mod-67.2) or FLAG epitope. "Input" is the input fraction (10% of lysate used for immunoprecipitation); "Output IP," the supernatant after immunoprecipitation; "IP," the immunoprecipitate. The names of alleles included in analysis are indicated above the figure. Analysis of transgenic lines was performed on the *y*^*2*^*sc*^*D1*^*ct*^*6*^; *su(Hw)*^*v*^*/ su(Hw)*^*e04061*^ background. All results were reproduced in three independent experiments.
(TIF)

S5 Fig Effect of Su(Hw) and its derivatives on the activity of the *gypsy* insulator in the *ct*^*6*^ and *sc*^*D1*^ alleles. (A) Schemes (not to scale) of the *ct*^*6*^ allele. The gray oval (Wme) indicates the wing margin enhancer controlling *cut* expression in the wings. The transcription start site indicated by arrowheads. The *gypsy* insertion is shown as a triangle with the black circle (Gy) marking Su(Hw) binding sites. The names of alleles included in analysis are under the photos showing the cut wing phenotype. (B) Scheme of the *yellow/ac/sc* region in *sc*^*D*^ allele. The coordinates of the AS-C region are as defined by Campuzano et al. (1985) [76]. The *gypsy* (*sc*^*D1*^) insertion is shown as a triangle with an arrow. Thick horizontal gray arrows show the positions and direction of *yellow* and AS-C gene transcripts. Filled oval indicate the endogenous Su(Hw) insulator. The standard nomenclature for each bristle is as follows: HU, humeral; AOR, anterior orbital; PS, presutural; ASA, anterior supra-alar; OC, ocellar; PV, postvertical; ANP, anterior notopleural; SC, scutellar. Only the bristles affected in the *ac* and *sc* mutations are shown. Empty boxes indicate that the corresponding bristles are present (wild-type phenotype). Filled boxes indicate the absence of the corresponding bristle(s) in more 90% of the flies.
(TIF)

S6 Fig The sequence of the oligonucleotide used to produce the synthetic region consisting of the Su(Hw) sites (S^×4^). This oligonucleotide contains *gypsy* insulator sequences corresponding to binding site 3 and includes the consensus sequence with three modules that represents the highest-affinity Su(Hw) binding site [57].
(TIF)

S7 Fig Examples of abdominals and eyes pigmentation. Photos represent the abdominal and eyes pigmentation in the 3-day-old males. Numbers indicate the scores of *yellow* expression in the abdominal segments, which ranged from 1 (pigmentation as in *y*^*1*^ allele) to 5 (pigmentation as in wild-type flies). Wild-type *white* expression determined the bright red eye color (R); in the absence of *white* expression, the eyes were white (W). Intermediate levels of pigmentation are yellow (Y), orange (Or), and brownish red (Br).
(TIF)

## References

[1] Lieberman-Aiden E, van Berkum NL, Williams L, Imakaev M, Ragoczy T, Telling A, et al Comprehensive mapping of long-range interactions reveals folding principles of the human genome. Science. 2009;326: 289–93. doi: 10.1126/science.1181369 19815776 10.1126/science.1181369 PMC2858594

[2] Dixon JR, Selvaraj S, Yue F, Kim A, Li Y, Shen Y, et al Topological domains in mammalian genomes identified by analysis of chromatin interactions. Nature. 2012;485: 376–80. doi: 10.1038/nature11082 22495300 10.1038/nature11082 PMC3356448

[3] Sexton T, Yaffe E, Kenigsberg E, Bantignies F, Leblanc B, Hoichman M, et al Three-dimensional folding and functional organization principles of the Drosophila genome. Cell. 2012;148: 458–72. doi: 10.1016/j.cell.2012.01.010 22265598 10.1016/j.cell.2012.01.010

[4] Ulianov SV, Khrameeva EE, Gavrilov AA, Flyamer IM, Kos P, Mikhaleva EA, et al Active chromatin and transcription play a key role in chromosome partitioning into topologically associating domains. Genome Res. 2016;26: 70–84. doi: 10.1101/gr.196006.115 26518482 10.1101/gr.196006.115 PMC4691752

[5] Ulianov SV, Tachibana-Konwalski K, Razin SV. Single-cell Hi-C bridges microscopy and genome-wide sequencing approaches to study 3D chromatin organization. Bioessays. 2017 doi: 10.1002/bies.201700104 28792605 10.1002/bies.201700104

[6] Dixon JR, Gorkin DU, Ren B. Chromatin Domains: The Unit of Chromosome Organization. Mol Cell. 2016;62: 668–80. doi: 10.1016/j.molcel.2016.05.018 27259200 10.1016/j.molcel.2016.05.018 PMC5371509

[7] Hnisz D, Day DS, Young RA. Insulated Neighborhoods: Structural and Functional Units of Mammalian Gene Control. Cell. 2016;167: 1188–200. doi: 10.1016/j.cell.2016.10.024 27863240 10.1016/j.cell.2016.10.024 PMC5125522

[8] Ulianov SV, Gavrilov AA, Razin SV. Nuclear compartments, genome folding, and enhancer-promoter communication. Int Rev Cell Mol Biol. 2015;315: 183–244. doi: 10.1016/bs.ircmb.2014.11.004 25708464 10.1016/bs.ircmb.2014.11.004

[9] Ghavi-Helm Y, Klein FA, Pakozdi T, Ciglar L, Noordermeer D, Huber W, et al Enhancer loops appear stable during development and are associated with paused polymerase. Nature. 2014;512: 96–100. doi: 10.1038/nature13417 25043061 10.1038/nature13417

[10] Fedotova AA, Bonchuk AN, Mogila VA, Georgiev PG. C2H2 Zinc Finger Proteins: The Largest but Poorly Explored Family of Higher Eukaryotic Transcription Factors. Acta Naturae. 2017;9: 47–58. 28740726 PMC5509000

[11] Maksimenko O, Georgiev P. Mechanisms and proteins involved in long-distance interactions. Front Genet. 2014;5: 28 doi: 10.3389/fgene.2014.00028 24600469 10.3389/fgene.2014.00028 PMC3927085

[12] Schwartz YB, Cavalli G. Three-Dimensional Genome Organization and Function in Drosophila. Genetics. 2017;205: 5–24. doi: 10.1534/genetics.115.185132 28049701 10.1534/genetics.115.185132 PMC5223523

[13] Ali T, Renkawitz R, Bartkuhn M. Insulators and domains of gene expression. Curr Opin Genet Dev. 2016;37: 17–26. doi: 10.1016/j.gde.2015.11.009 26802288 10.1016/j.gde.2015.11.009

[14] Chetverina D, Fujioka M, Erokhin M, Georgiev P, Jaynes JB, Schedl P. Boundaries of loop domains (insulators): Determinants of chromosome form and function in multicellular eukaryotes. Bioessays. 2017;39(3). 10.1002/bies.201600233 PMC5536339 28133765

[15] Kyrchanova O, Georgiev P. Chromatin insulators and long-distance interactions in Drosophila. FEBS Lett. 2014;588: 8–14. doi: 10.1016/j.febslet.2013.10.039 24211836 10.1016/j.febslet.2013.10.039

[16] Matzat LH, Lei EP. Surviving an identity crisis: a revised view of chromatin insulators in the genomics era. Biochim Biophys Acta. 2014;1839: 203–14. doi: 10.1016/j.bbagrm.2013.10.007 24189492 10.1016/j.bbagrm.2013.10.007 PMC3951628

[17] Comet I, Savitskaya E, Schuettengruber B, Negre N, Lavrov S, et al PRE-mediated bypass of two Su(Hw) insulators targets PcG proteins to a downstream promoter. Dev Cell. 2006;11: 117–124. doi: 10.1016/j.devcel.2006.05.009 16824958 10.1016/j.devcel.2006.05.009

[18] Fujioka M, Mistry H, Schedl P, Jaynes JB. Determinants of Chromosome Architecture: Insulator Pairing in cis and in trans. PLoS Genet. 2016;12: e1005889 doi: 10.1371/journal.pgen.1005889 26910731 10.1371/journal.pgen.1005889 PMC4765946

[19] Kyrchanova O, Chetverina D, Maksimenko O, Kullyev A, Georgiev P. Orientation-dependent interaction between Drosophila insulators is a property of this class of regulatory elements. Nucleic Acids Res. 2008;36: 7019–7028. doi: 10.1093/nar/gkn781 18987002 10.1093/nar/gkn781 PMC2602758

[20] Kyrchanova O, Ivlieva T, Toshchakov S, Parshikov A, Maksimenko O, et al Selective interactions of boundaries with upstream region of Abd-B promoter in Drosophila bithorax complex and role of dCTCF in this process. Nucleic Acids Res. 2011;39: 3042–3052. doi: 10.1093/nar/gkq1248 21149269 10.1093/nar/gkq1248 PMC3082887

[21] Kyrchanova O, Mogila V, Wolle D, Deshpande G, Parshikov A, et al Functional Dissection of the Blocking and Bypass Activities of the Fab-8 Boundary in the Drosophila Bithorax Complex. PLoS Genet. 2016;12: e1006188 doi: 10.1371/journal.pgen.1006188 27428541 10.1371/journal.pgen.1006188 PMC4948906

[22] Zolotarev N, Fedotova A, Kyrchanova O, Bonchuk A, Penin AA, et al Architectural proteins Pita, Zw5,and ZIPIC contain homodimerization domain and support specific long-range interactions in Drosophila. Nucleic Acids Res. 2016;44: 7228–7241. doi: 10.1093/nar/gkw371 27137890 10.1093/nar/gkw371 PMC5009728

[23] Ahanger SH, Shouche YS, Mishra RK. Functional sub-division of the Drosophila genome via chromatin looping: the emerging importance of CP190. Nucleus. 2013;4: 115–22. doi: 10.4161/nucl.23389 23333867 10.4161/nucl.23389 PMC3621743

[24] Chetverina D, Aoki T, Erokhin M, Georgiev P, Schedl P. Making connections: insulators organize eukaryotic chromosomes into independent cis-regulatory networks. Bioessays. 2014;36: 163–72. doi: 10.1002/bies.201300125 24277632 10.1002/bies.201300125 PMC4362772

[25] Ghirlando R, Felsenfeld G. CTCF: making the right connections. Genes Dev. 2016;30: 881–91. doi: 10.1101/gad.277863.116 27083996 10.1101/gad.277863.116 PMC4840295

[26] Kyrchanova O, Mogila V, Wolle D, Magbanua JP, White R, Georgiev P, et al The boundary paradox in the Bithorax complex. Mech Dev. 2015;138: 122–32. doi: 10.1016/j.mod.2015.07.002 26215349 10.1016/j.mod.2015.07.002 PMC4890074

[27] Razin SV, Borunova VV, Maksimenko OG, Kantidze OL. Cys2His2 zinc finger protein family: classification, functions, and major members. Biochemistry (Mosc). 2012;77: 217–26. 22803940 10.1134/S0006297912030017

[28] Heger P, George R, Wiehe T. Successive gain of insulator proteins in arthropod evolution. Evolution. 2013;67: 2945–56. doi: 10.1111/evo.12155 24094345 10.1111/evo.12155 PMC4208683

[29] Pauli T, Vedder L, Dowling D, Petersen M, Meusemann K, Donath A, et al Transcriptomic data from panarthropods shed new light on the evolution of insulator binding proteins in insects: Insect insulator proteins. BMC Genomics. 2016;17: 861 doi: 10.1186/s12864-016-3205-1 27809783 10.1186/s12864-016-3205-1 PMC5094011

[30] Pavletich NP, Pabo CO. Zinc finger-DNA recognition: crystal structure of a Zif268-DNA complex at 2.1 A. Science. 1991;252: 809–17. 2028256 10.1126/science.2028256

[31] Persikov AV, Singh M. De novo prediction of DNA-binding specificities for Cys2His2 zinc finger proteins. Nucleic Acids Res. 2014;42: 97–108. doi: 10.1093/nar/gkt890 24097433 10.1093/nar/gkt890 PMC3874201

[32] Iuchi S. Three classes of C2H2 zinc finger proteins. Cell Mol Life Sci. 2001;58: 625–35. doi: 10.1007/PL00000885 11361095 10.1007/PL00000885 PMC11146492

[33] Saldana-Meyer R, Gonzalez-Buendia E, Guerrero G, Narendra V, Bonasio R, Recillas-Targa F, et al CTCF regulates the human p53 gene through direct interaction with its natural antisense transcript, Wrap53. Genes Dev. 2014;28: 723–34. doi: 10.1101/gad.236869.113 24696455 10.1101/gad.236869.113 PMC4015496

[34] Negre N, Brown CD, Shah PK, Kheradpour P, Morrison CA, Henikoff JG, et al A comprehensive map of insulator elements for the Drosophila genome. PLoS Genet. 2010;6: e1000814 doi: 10.1371/journal.pgen.1000814 20084099 10.1371/journal.pgen.1000814 PMC2797089

[35] Schwartz YB, Linder-Basso D, Kharchenko PV, Tolstorukov MY, Kim M, Li HB, et al Nature and function of insulator protein binding sites in the Drosophila genome. Genome Res. 2012;22: 2188–98. doi: 10.1101/gr.138156.112 22767387 10.1101/gr.138156.112 PMC3483548

[36] Scott KC, Taubman AD, Geyer PK. Enhancer blocking by the Drosophila gypsy insulator depends upon insulator anatomy and enhancer strength. Genetics. 1999;153: 787–98. 10511558 10.1093/genetics/153.2.787 PMC1460797

[37] Gause M, Morcillo P, Dorsett D. Insulation of enhancer-promoter communication by a gypsy transposon insert in the Drosophila cut gene: cooperation between suppressor of hairy-wing and modifier of mdg4 proteins. Mol Cell Biol. 2001;21: 4807–17. doi: 10.1128/MCB.21.14.4807-4817.2001 11416154 10.1128/MCB.21.14.4807-4817.2001 PMC87172

[38] Georgiev P, Kozycina M. Interaction between mutations in the suppressor of Hairy wing and modifier of mdg4 genes of Drosophila melanogaster affecting the phenotype of gypsy-induced mutations. Genetics. 1996;142: 425–36. 8852842 10.1093/genetics/142.2.425 PMC1206977

[39] Gerasimova TI, Gdula DA, Gerasimov DV, Simonova O, Corces VG. A Drosophila protein that imparts directionality on a chromatin insulator is an enhancer of position-effect variegation. Cell. 1995;82: 587–97. 7664338 10.1016/0092-8674(95)90031-4

[40] Ghosh D, Gerasimova TI, Corces VG. Interactions between the Su(Hw) and Mod(mdg4) proteins required for gypsy insulator function. EMBO J. 2001;20: 2518–27. doi: 10.1093/emboj/20.10.2518 11350941 10.1093/emboj/20.10.2518 PMC125459

[41] Golovnin A, Mazur A, Kopantseva M, Kurshakova M, Gulak PV, Gilmore B, et al Integrity of the Mod(mdg4)-67.2 BTB domain is critical to insulator function in Drosophila melanogaster. Mol Cell Biol. 2007;27: 963–74. doi: 10.1128/MCB.00795-06 17101769 10.1128/MCB.00795-06 PMC1800699

[42] Kurshakova M, Maksimenko O, Golovnin A, Pulina M, Georgieva S, Georgiev P, et al Evolutionarily conserved E(y)2/Sus1 protein is essential for the barrier activity of Su(Hw)-dependent insulators in Drosophila. Mol Cell. 2007;27: 332–8. doi: 10.1016/j.molcel.2007.05.035 17643381 10.1016/j.molcel.2007.05.035

[43] Pai CY, Lei EP, Ghosh D, Corces VG. The centrosomal protein CP190 is a component of the gypsy chromatin insulator. Mol Cell. 2004;16: 737–48. doi: 10.1016/j.molcel.2004.11.004 15574329 10.1016/j.molcel.2004.11.004

[44] Alekseyenko AA, Gorchakov AA, Zee BM, Fuchs SM, Kharchenko PV, Kuroda MI. Heterochromatin-associated interactions of Drosophila HP1a with dADD1, HIPP1, and repetitive RNAs. Genes Dev. 2014;28: 1445–60. doi: 10.1101/gad.241950.114 24990964 10.1101/gad.241950.114 PMC4083088

[45] King MR, Matzat LH, Dale RK, Lim SJ, Lei EP. The RNA-binding protein Rumpelstiltskin antagonizes gypsy chromatin insulator function in a tissue-specific manner. J Cell Sci. 2014;127: 2956–66. doi: 10.1242/jcs.151126 24706949 10.1242/jcs.151126 PMC4075359

[46] Matzat LH, Dale RK, Moshkovich N, Lei EP. Tissue-specific regulation of chromatin insulator function. PLoS Genet. 2012;8: e1003069 doi: 10.1371/journal.pgen.1003069 23209434 10.1371/journal.pgen.1003069 PMC3510032

[47] Bonchuk A, Denisov S, Georgiev P, Maksimenko O. Drosophila BTB/POZ domains of "ttk group" can form multimers and selectively interact with each other. J Mol Biol. 2011;412: 423–36. doi: 10.1016/j.jmb.2011.07.052 21821048 10.1016/j.jmb.2011.07.052

[48] Oliver D, Sheehan B, South H, Akbari O, Pai CY. The chromosomal association/dissociation of the chromatin insulator protein Cp190 of Drosophila melanogaster is mediated by the BTB/POZ domain and two acidic regions. BMC Cell Biol. 2010;11: 101 doi: 10.1186/1471-2121-11-101 21194420 10.1186/1471-2121-11-101 PMC3022720

[49] Vogelmann J, Le Gall A, Dejardin S, Allemand F, Gamot A, Labesse G, et al Chromatin insulator factors involved in long-range DNA interactions and their role in the folding of the Drosophila genome. PLoS Genet. 2014;10: e1004544 doi: 10.1371/journal.pgen.1004544 25165871 10.1371/journal.pgen.1004544 PMC4148193

[50] Plevock KM, Galletta BJ, Slep KC, Rusan NM. Newly Characterized Region of CP190 Associates with Microtubules and Mediates Proper Spindle Morphology in Drosophila Stem Cells. PLoS One. 2015;10: e0144174 doi: 10.1371/journal.pone.0144174 26649574 10.1371/journal.pone.0144174 PMC4674064

[51] Bonchuk A, Maksimenko O, Kyrchanova O, Ivlieva T, Mogila V, Deshpande G, et al Functional role of dimerization and CP190 interacting domains of CTCF protein in Drosophila melanogaster. BMC Biol. 2015;13: 63 doi: 10.1186/s12915-015-0168-7 26248466 10.1186/s12915-015-0168-7 PMC4528719

[52] Bartkuhn M, Straub T, Herold M, Herrmann M, Rathke C, Saumweber H, et al Active promoters and insulators are marked by the centrosomal protein 190. EMBO J. 2009;28: 877–88. doi: 10.1038/emboj.2009.34 19229299 10.1038/emboj.2009.34 PMC2670862

[53] Cuartero S, Fresan U, Reina O, Planet E, Espinas ML. Ibf1 and Ibf2 are novel CP190-interacting proteins required for insulator function. EMBO J. 2014;33: 637–47. doi: 10.1002/embj.201386001 24502977 10.1002/embj.201386001 PMC3989656

[54] Maksimenko O, Bartkuhn M, Stakhov V, Herold M, Zolotarev N, Jox T, et al Two new insulator proteins, Pita and ZIPIC, target CP190 to chromatin. Genome Res. 2015;25: 89–99. doi: 10.1101/gr.174169.114 25342723 10.1101/gr.174169.114 PMC4317163

[55] Mohan M, Bartkuhn M, Herold M, Philippen A, Heinl N, Bardenhagen I, et al The Drosophila insulator proteins CTCF and CP190 link enhancer blocking to body patterning. The EMBO J. 2007;26: 4203–14. doi: 10.1038/sj.emboj.7601851 17805343 10.1038/sj.emboj.7601851 PMC2230845

[56] Soshnev AA, He B, Baxley RM, Jiang N, Hart CM, Tan K, et al Genome-wide studies of the multi-zinc finger Drosophila Suppressor of Hairy-wing protein in the ovary. Nucleic Acids Res. 2012;40: 5415–31. doi: 10.1093/nar/gks225 22406832 10.1093/nar/gks225 PMC3384341

[57] Baxley RM, Bullard JD, Klein MW, Fell AG, Morales-Rosado JA, Duan T, et al Deciphering the DNA code for the function of the Drosophila polydactyl zinc finger protein Suppressor of Hairy-wing. Nucleic Acids Res. 2017 doi: 10.1093/nar/gkx040 28158673 10.1093/nar/gkx040 PMC5416891

[58] Buxa MK, Slotman JA, van Royen ME, Paul MW, Houtsmuller AB, Renkawitz R. Insulator speckles associated with long-distance chromatin contacts. Biol Open. 2016;5: 1266–74. doi: 10.1242/bio.019455 27464669 10.1242/bio.019455 PMC5051650

[59] Golovnin A, Melnikova L, Volkov I, Kostuchenko M, Galkin AV, Georgiev P. 'Insulator bodies' are aggregates of proteins but not of insulators. EMBO Rep. 2008;9: 440–5. doi: 10.1038/embor.2008.32 18369369 10.1038/embor.2008.32 PMC2373365

[60] Golovnin A, Volkov I, Georgiev P. SUMO conjugation is required for the assembly of Drosophila Su(Hw) and Mod(mdg4) into insulator bodies that facilitate insulator complex formation. J Cell Sci. 2012;125: 2064–74. doi: 10.1242/jcs.100172 22375064 10.1242/jcs.100172

[61] Melnikova L, Shapovalov I, Kostyuchenko M, Georgiev P, Golovnin A. EAST affects the activity of Su(Hw) insulators by two different mechanisms in Drosophila melanogaster. Chromosoma. 2017;126: 299–311. doi: 10.1007/s00412-016-0596-3 27136940 10.1007/s00412-016-0596-3

[62] Schoborg T, Rickels R, Barrios J, Labrador M. Chromatin insulator bodies are nuclear structures that form in response to osmotic stress and cell death. J Cell Biol. 2013;202: 261–76. doi: 10.1083/jcb.201304181 23878275 10.1083/jcb.201304181 PMC3718971

[63] Harrison DA, Gdula DA, Coyne RS, Corces VG. A leucine zipper domain of the suppressor of Hairy-wing protein mediates its repressive effect on enhancer function. Genes Dev. 1993;7: 1966–78. 7916729 10.1101/gad.7.10.1966

[64] Karess RE, Rubin GM. Analysis of P transposable element functions in Drosophila. Cell. 1984;38: 135–46. 6088058 10.1016/0092-8674(84)90534-8

[65] Baxley RM, Soshnev AA, Koryakov DE, Zhimulev IF, Geyer PK. The role of the Suppressor of Hairy-wing insulator protein in Drosophila oogenesis. Dev Biol. 2011;356: 398–410. doi: 10.1016/j.ydbio.2011.05.666 21651900 10.1016/j.ydbio.2011.05.666 PMC3143288

[66] Bischof J, Maeda RK, Hediger M, Karch F, Basler K. An optimized transgenesis system for Drosophila using germ-line-specific phiC31 integrases. Proc Natl Acad Sci U S A. 2007;104: 3312–7. doi: 10.1073/pnas.0611511104 17360644 10.1073/pnas.0611511104 PMC1805588

[67] Panteleev D, Kakpakov VT, Gorelova TV, Andrianov BV. [Variability of continuous insect cell lines and their identification]. Tsitologiia. 2006;48: 653–60. 17147256

[68] Schneider I. Cell lines derived from late embryonic stages of Drosophila melanogaster. J Embryol Exp Morphol. 1972;27: 353–65. 4625067

[69] Georgieva SG, Nabirochkina EN, Ladygina NG, Georgiev PG, Soldatov AV. [Nuclear protein e(y)2 from Drosophila melanogaster participates in transcription control]. Genetika. 2001;37: 24–8. 11234421

[70] Melnikova L, Kostyuchenko M, Molodina V, Parshikov A, Georgiev P, Golovnin A. Multiple interactions are involved in a highly specific association of the Mod(mdg4)-67.2 isoform with the Su(Hw) sites in Drosophila. Open Biol. 2017; pii: 170150. doi: 10.1098/rsob.170150 29021216 10.1098/rsob.170150 PMC5666082

[71] Melnikova L, Kostyuchenko M, Molodina V, Parshikov A, Georgiev P, Golovnin A. Interactions between BTB domain of CP190 and two adjacent regions in Su(Hw) are required for the insulator complex formation. Chromosoma. 2017 doi: 10.1007/s00412-017-0645-6 28939920 10.1007/s00412-017-0645-6

[72] Mazur AM, Georgiev PG, Golovnin AK. The acid domain located at the C-terminus of the Su(Hw) protein represses transcription in the yeast two-hybrid system. Dokl Biochem Biophys. 2005;400: 1–3. 15846971 10.1007/s10628-005-0018-6

[73] Kuhn-Parnell EJ, Helou C, Marion DJ, Gilmore BL, Parnell TJ, Wold MS, et al Investigation of the properties of non-gypsy suppressor of hairy-wing-binding sites. Genetics. 2008;179: 1263–73. doi: 10.1534/genetics.108.087254 18562648 10.1534/genetics.108.087254 PMC2475731

[74] Geyer PK, Spana C, Corces VG. On the molecular mechanism of gypsy-induced mutations at the yellow locus of Drosophila melanogaster. EMBO J. 1986;5: 2657–62. 3096713 10.1002/j.1460-2075.1986.tb04548.x PMC1167166

[75] Geyer PK, Corces VG. Separate regulatory elements are responsible for the complex pattern of tissue-specific and developmental transcription of the yellow locus in Drosophila melanogaster. Genes Dev. 1987;1: 996–1004. 3123324 10.1101/gad.1.9.996

[76] Campuzano S, Carramolino L, Cabrera CV, Ruiz-Gomez M, Villares R, Boronat A, et al Molecular genetics of the achaete-scute gene complex of D. melanogaster. Cell. 1985;40: 327–38. 3917860 10.1016/0092-8674(85)90147-3

[77] Qian S, Varjavand B, Pirrotta V. Molecular analysis of the zeste-white interaction reveals a promoter-proximal element essential for distant enhancer-promoter communication. Genetics. 1992;131: 79–90. 1375573 10.1093/genetics/131.1.79 PMC1204967

[78] Mallin DR, Myung JS, Patton JS, Geyer PK. Polycomb group repression is blocked by the Drosophila suppressor of Hairy-wing [su(Hw)] insulator. Genetics. 1998;148: 331–9. 9475743 10.1093/genetics/148.1.331 PMC1459791

[79] Sigrist CJ, Pirrotta V. Chromatin insulator elements block the silencing of a target gene by the Drosophila polycomb response element (PRE) but allow trans interactions between PREs on different chromosomes. Genetics. 1997;147: 209–21. 9286681 10.1093/genetics/147.1.209 PMC1208105

[80] Kassis JA, Kennison JA, Tamkun JW. Polycomb and Trithorax Group Genes in Drosophila. Genetics. 2017;206: 1699–725. doi: 10.1534/genetics.115.185116 28778878 10.1534/genetics.115.185116 PMC5560782

[81] Gerasimova TI, Byrd K, Corces VG. A chromatin insulator determines the nuclear localization of DNA. Mol Cell. 2000;6: 1025–35. 11106742 10.1016/s1097-2765(00)00101-5

[82] Golovnin A, Melnikova L, Shapovalov I, Kostyuchenko M, Georgiev P. EAST Organizes Drosophila Insulator Proteins in the Interchromosomal Nuclear Compartment and Modulates CP190 Binding to Chromatin. PLoS One. 2015;10: e0140991 doi: 10.1371/journal.pone.0140991 26489095 10.1371/journal.pone.0140991 PMC4638101

[83] Rath U, Wang D, Ding Y, Xu YZ, Qi H, et al Chromator, a novel and essential chromodomain protein interacts directly with the putative spindle matrix protein skeletor. J Cell Biochem. 2004;93: 1033–1047. doi: 10.1002/jcb.20243 15389869 10.1002/jcb.20243

[84] Gdula DA, Corces VG. Characterization of functional domains of the su(Hw) protein that mediate the silencing effect of mod(mdg4) mutations. Genetics. 1997;145: 153–161. 9017397 10.1093/genetics/145.1.153 PMC1207773

