# Supplementary material for: Role of Su(Hw) zinc finger 10 and interaction with CP190 and Mod(mdg4) proteins in recruiting the Su(Hw) complex to chromatin sites in Drosophila
Source: PLoS One. 2018 Feb 23;13(2):e0193497. doi: 10.1371/journal.pone.0193497 (PMC5825117; doi:10.1371/journal.pone.0193497)
Supplement: S1 Appendix — (DOCX) [file pone.0193497.s001.docx]

**Phenotypic analysis of effect of different Su(Hw) variants on the activity of *gypsy* insulator in *cut* and *scute* loci**

The effects of various mutation combinations were scored by two researchers independently. The *cut^6^* phenotype was scored in 3- to 5-day-old males. Representative wing forms shown in the figures were selected as “average” from the series of wings. In the *sc^D1^* allele, the formation of several bristles was affected: the humeral (HU), anterior orbital (AOR), presutural (PS), anterior supra-alar (ASA), ocellar (OC), postvertical (PV), anterior notopleural (ANP), scutellar (SC) bristles failed to develop in more 90% of the examined flies. Changes in the *sc^D1^* and *cut^6^* phenotypes in combinations with different Su(Hw) variants were scored in at least 50 flies from each line. The effects of *su(Hw)* and *mod(mdg4)^u1^* mutations on *sc^D1^* and *cut^6^*  were tested as described [1].

**Protein extract preparation and co-IP** **analysis**

The material (about 150–200 mg of adult male flies, sufficient for four to five independent immunoprecipitations) was homogenized in 5 ml of buffer IP-S+ (10 mM Tris-HCl, pH 7.5; 10 mM NaCl, 10 mM MgCl_2_, 1 mM EDTA, 1 mM EGTA, 1 mM DTT, and 250 mM sucrose with PMSF, leupeptin, and pepstatin A) at 4°C using a Dounce homogenizer with type A pestle. The homogenate was filtered through a BD Falcon filter into a 50-mL tube and pelleted by centrifugation at 4000 *g,* 4°C for 5 min. The supernatant was discarded, and the pellet was washed with three 3-mL portions of buffer IP-S+, with pelleting at 4000 g for 5 min after each wash. The final pellet was resuspended in 0.5 mL of IP-10+ buffer **(**10 mM Tris-HCl, pH 7.5; 10 mM NaCl, 10 mM MgCl_2_, 1 mM EDTA, 1 mM EGTA, 1mM DTT, 0,1% NP-40, 10% glycerol, and Roche Complete Protease Inhibitor Cocktail) and homogenized at 4°C using a Dounce homogenizer with type B pestle. The homogenate was supplemented with an equal volume of IP-850 buffer (10 mM Tris-HCl, pH 7.5; 850 mM NaCl, 10 mM MgCl_2_, 1 mM EDTA, 1 mM EGTA, 1 mM DTT, 0,1% NP-40, 10% glycerol, and Roche Complete Protease Inhibitor Cocktail), mixed gently, and left on ice for 30–60 min. This was followed by 4 rounds of centrifugation at 14 000 g for 15 min, with the supernatant being each time transferred to a new tube without disturbing the pellet. Immediately before experiments, the lysate was diluted with three volumes of IP-0 buffer **(**10 mM Tris-HCl, pH 7.5; 10 mM MgCl_2_, 1 mM EDTA, 1 mM EGTA, 1 mM DTT, 0.1% NP-40, 10% glycerol, and Roche Complete Protease Inhibitor Cocktail), centrifuged as above, and transferred to a new tube. Co-IP analysis was performed as described [2].

**Constructs for yeast two-hybrid assay**

The coding region of Mod(mdg4)-67.2 from pGEX2T (kindly provided by D. Dorsett) was cloned in vectors pGBT9 or pGDA (plasmid that allows producing fusion proteins with the Gal4 activation domain in the C-terminal part) using *Eco*RI and *Bam*HI sites (**pGBT9 Mod-67.2 and pGDAMod-67.2**).

To generate **pGDA CP-190** and **pGBT9 CP-190**, the corresponding fragment from CP190 cDNA was PCR-amplified with primers 5'-catgggtgaagtcaagtc-3' and 5'-ttcagatctttccaggttgtcaatgg-3' containing *Bgl*II site. This PCR product was cloned in either pGDA or pGBT9 cleaved with *Eco*RI (filled in with Klenow fragment) and *Bam*HI.

To generate **pGBT9 Su(Hw)** plasmid, Su(Hw) cDNA was PCR-amplified from pGEM3Zf Su(Hw) (kindly provided by D. Dorsett) with primers 5'-aatgagtgcctccaaggagggc-3' (upstream) and 5'-ccgtcgactcaagctttctcttgttc-3' (downstream, containing *Sal*I site). The PCR product was digested with *Sal*I and cloned in the pGBT9 vector cleaved with *Sma*I and *Sal*I.

To generate **pGDA Su(Hw)** plasmid, Su(Hw) cDNA was PCR-amplified from pGEM3Zf Su(Hw) with primers 5'-aatgagtgcctccaaggagggc-3' (upstream) and 5'-aaccatggaagctttctcttgttc-3' (downstream, containing *Nco*I site). This PCR product was digested with *Nco*I and cloned in pGDA cleaved with *Hpa*I and *Nco*I.

To generate **pGBT9 Su(Hw)^ΔC^** plasmid, Su(Hw) lacking the C-end (1–892) was PCR-amplified as described above, but the next downstream primer was 5'-tttgtcgacttcgcctgtgac-3' (with *Sal*I site). The PCR product was digested with *Sal*I and cloned in pGBT9 cleaved with *Sma*I and *Sal*I.

To generate **pGDA Su(Hw)^ΔC^** plasmid, we used the same strategy, but the next downstream primer was 5'-tttccatggttcgcctgtgac-3' (with *Nco*I site).

To generate **pGBT9 Su(Hw)^Δ10^** plasmid, Su(Hw) cDNA was PCR-amplified from pGEM3Zf Su(Hw) with primers 5’-gcgaatatgggttttggaatgg-3’/5’-ggaacagcacaagtcacgtg-3’ (upstream primer) and 5’-gtcttttcctgcgccgagtg-3’/5’-catcgccttcgtccacgag-3’ (downstream primer). Both PCR products were treated with kinase enzyme, and then the first one was digested with *Bgl*II, and the second one, with *Eag*I. The resulting fragments were simultaneously cloned in pGBT9 Su(Hw)^ΔC^ plasmid digested with *Bgl*II and *Eag*I. The deletion was verified by sequencing.

To generate **pGDA Su(Hw)^Δ10^** plasmid, an *X*baI–*Eag*I fragment from pGBT9 Su(Hw)^Δ10^ plasmid was cloned to pGDA Su(Hw)^ΔC^.

To generate **pGBT9 Su(Hw)^f^** plasmid, Su(Hw) cDNA was PCR-amplified from *su(Hw)^f^* mutant genome DNA with primers 5’-gcgaatatgggttttggaatgg-3’/5’-ggaacagcacaagtcacgtg-3’ (upstream primer) and 5’-ccctttccctgtgagcaatgc-3’/5’-catcgccttcgtccacgag-3’ (downstream primer). Both PCR products were treated with kinase enzyme, and then the first product was digested with *Bgl*II, and the second one, with *Eag*I. The resulting fragments were simultaneously cloned in pGBT9 Su(Hw)^ΔC^ plasmid digested with *Bgl*II and *Eag*I, and the deletion was verified by sequencing.

To generate **pGDA Su(Hw)^f^** plasmid, an *X*baI–*Eag*I fragment from pGBT9 Su(Hw)^Δ10^ plasmid was cloned to pGDA Su(Hw)^ΔC^.

**Transgenic constructs**

To prepare **pAc5.1 FLAG-Su(Hw)^+^**, the *Xho*I–*Hind*III (filled in with Klenow) fragment from pGem3 Su(Hw) (kindly provided by D. Dorsett) was cloned in the corresponding vector at *Xho*I and *BamH*I (filled in with Klenow) sites.

To generate **pAc5.1 FLAG-Su(Hw)^f^** or **pAc5.1 FLAG-Su(Hw)^Δ10^**, an *Xba*I–*EcoR*I fragment either from pGBT9 Su(Hw)^f^ or from pGBT9 Su(Hw)^Δ10^ was cloned in pAc5.1 FLAG-Su(Hw)^+^ plasmid digested with *Xba*I and *Eco*RI.

To express the transgenic constructs in flies, we generated vector **pUbqW** with *ubiquitin-63E* promoter and *miniwhite* gene as a marker. To prepare pUbqW, the fragment containing SV40 terminator from pUAST plasmid was recovered with *Xba*I and *Bam*HI (both filled in with Klenow fragment) and cloned in pCaSpeR4 cleaved with *Pst*I filled in with Klenow fragment (pCaSpeR4-SV40). The correct orientation of the fragment was verified by sequencing. Then a 1868-bp promoter region was PCR-amplified with primers 5'-agaattctgtcgccggaacgcag-3' and 5'-agaattcgcattttggattattctgcggg-3', both containing *Eco*RI site. The PCR product was recovered with *Eco*RI and cloned in pCaSpeR4-SV40 cleaved with the same enzyme. Its correct orientation was verified by sequencing.

To express the transgenic constructs in flies, we generated vector **pWAB** with the Actin 5C promoter, att*B* site for phiC31-based integration, and *miniwhite* gene as a marker. First, the att*B* site was attached to the *miniwhite* gene by their sequential cloning site in pBluescript II SK(+) vector (pSK). To prepare pWAB, fragment containing the *miniwhite* gene with the attached att*B* site was recovered from pSK *miniwite*–att*B* plasmid by *Eco*RI enzyme, filled in with Klenow fragment and cloned in pAc5.1-FLAG vector digested with *H*paI.

To generate the ***P{w^+^;WAB-Su(Hw)1-945-FLAG}*** construct, the *Eco*RI–*Eco*RII fragment containing 2014bp cDNA of Su(Hw) protein was cloned in pWAB vector cleaved with *Eco*RI. At the next step, the C-terminal fragment of Su(Hw) digested first with *Not*I (filled in with Klenow fragment) and then with *Eag*I was cloned in obtained plasmid digested first with *BamH*I (filled in with Klenow fragment) and then with *Eag*I.

To generate the ***P{w^+^;WAB-Su(Hw)^Δ10^ -FLAG}*** construct, the *EcoR*I–*EcoRI*I fragment from pGBT9 Su(Hw)^Δ10^ containing 2014-bp cDNA of Su(Hw) was cloned in pWAB vector cleaved with *Eco*RI. The C-terminal fragment of Su(Hw) digested with *Not*I (filled in with Klenow fragment) and *Eag*I was then cloned in the obtained plasmid digested first by *BamH*I (filled in with Klenow fragment) and then with *Eag*I.

To generate the ***P{w^+^;WAB-Su(Hw)^f^ -FLAG}*** construct, the *EcoR*I–*EcoRI*I fragment from pGBT9 Su(Hw)^f^ containing 2014-bp cDNA Su(Hw) was cloned in pWAB vector cleaved with *Eco*RI enzyme. The C-terminal fragment of Su(Hw) digested with *Not*I (filled in with Klenow fragment) and *Eag*I was then cloned in the obtained plasmid digested first by *BamH*I (filled in with Klenow fragment) and then with *Eag*I.

To generate the ***P{w^+^;UbqW-Su(Hw)^f^ -FLAG}*** construct, the KpnI–SacI fragment (filled in with Klenow fragment) containing full-length cDNA of Su(Hw)^f^ mutant protein fused to FLAG epitope was cloned in pUbqW cleaved with *Kpn*I and *Bam*HI (filled in with Klenow fragment).

The 8-kb fragment containing the *yellow* gene and the cDNA *yellow* clone were kindly provided by P. Geyer. The 3-kb *Sal*I–*Bam*HI fragment containing the *yellow* regulatory region (yr) was subcloned into pGEM7 cleaved with *Bam*HI + *Xho*I (yr plasmid).

The 430-bp *gypsy* sequence containing the Su(Hw) binding region (Gy) was PCR-amplified from the *gypsy* retrotransposon (5’-ctggccacgtaataagtgtgcg-3’ and 5’-gttgttggttggcacaccacaaa-3’). After sequencing to confirm its identity, the product was inserted in the CaSpeR2 vector (C2-su). The 5-kb *Bam*HI–*Bgl*II fragment containing the *yellow* coding region (yc) was subcloned into CaSpeR3 (C3-yc) or CaSpeR2-su (C3-su-yc).

To generate the four (S^×4^) reiterated Su(Hw) binding sites, oligonucliotieds 5’-aaaaataagtgctgcatactttttagagaa-3’ and 5’-ttctctaaaaagtatgcagcacttattttt-3’ were annealed, treated with kinase, and ligated to pSK vector. Insertion with unidirectional orientation was selected for further study.

A 661-bp *Nde*I–*Pst*I fragment (PRE) containing the *Ubx* PRE sequence was kindly provided by V. Pirrotta. This fragment was inserted in the yr at position –1629 from the *yellow* start site (yr-PRE). The *white* regulatory sequences from position –1084 to –1465 bp relative to the transcription start site (Ee) contained the testis and eye enhancers. The Ee fragment was inserted in the yr at position –1629 relative to the *yellow* transcription start site, between the wing and body enhancers (yr-Ee).

**Eye(Gy)YW** and **Eye(S^×4^)YW**: The Gy, and S^×4^ fragments were cloned between the *loxP* sites (lox(Gy), and lox(S^×4^)). The resulting lox(Gy), and lox(S^×4^) fragments were inserted into the yr-Ee plasmid treated with *Eco*47III (yr-Ee–lox(Gy), and yr-Ee–lox(S^×4^)). The yr-Ee–lox(Gy), and yr-Ee–lox(S^×4^) fragments were ligated into C3-su-yc treated with *Xba*I and *Bam*HI*.*

**PRE(Gy)YW**, and **PRE(S^×4^)YW**: The lox(Gy), lox(S^×8^) and lox(S^×4^) fragments were inserted in the yr-PRE plasmid treated with *Eco*47III (yr-PRE–lox(Gy), yr-PRE–lox(S^×8^) and yr-PRE–lox(S^×4^)). The yr-PRE–lox(Gy), yr-PRE–lox(S^×8^) and yr-PRE–lox(S^×4^) fragments were ligated into C3-su-yc treated with *Xba*I and *Bam*HI*.*

# References

1. Georgiev P, Kozycina M (1996) Interaction between mutations in the suppressor of Hairy wing and modifier of mdg4 genes of Drosophila melanogaster affecting the phenotype of gypsy-induced mutations. Genetics 142:425-436

2. Georgieva SG, Nabirochkina EN, Ladigina NG, Georgiev PG, Soldatov AV (2001) The involvement of the Drosophila melanogaster nuclear protein e(y)2 in transcription regulation. Russian Journal of Genetics 37 (1):19-23
