# Supplementary material for: Role of Su(Hw) zinc finger 10 and interaction with CP190 and Mod(mdg4) proteins in recruiting the Su(Hw) complex to chromatin sites in Drosophila
Source: PLoS One. 2018 Feb 23;13(2):e0193497. doi: 10.1371/journal.pone.0193497 (PMC5825117; doi:10.1371/journal.pone.0193497)
Supplement: S1 Table — (DOC) [file pone.0193497.s002.doc]

**S1 Table.** Primer sequences used in PCR for ChIP analysis

| **Primer pair** | **Sequences** |
| --- | --- |
| 62D fw  62D rev | 5' TTTGGGCTTGGTGAGAACAG 3'  5' TGATACCAGGCGAACAGAAATC 3' |
| 50A fw  50A rev | 5' ATACAAAGTGGTTTCAGCCAAGAAG 3'  5' TTGATAAATAGTCCAGCACGCATAC 3' |
| 87E fw  87E rev | 5' GGATGTTACA TTGAGAGTGCTTAGG 3'  5' TTTGCGTTTCGGCTGCTGTC 3' |
| 1A2 fw  1A2 rev | 5' ACCACACATCAGTCATCGTGT 3'  5' CTTCGTCTACCGTTGTGC 3' |
| Gypsy fw  Gypsy rev | 5' TTCTCTAAAAAGTATGCAGCACTT 3'  5' CACGTAATAAGTGTGCGTTGA 3' |
| 66E fw  66E rev | 5' GCTGCTGATCCTCGCTTTCC 3'  5' AACTCCATTCCATTCACCTGTCTC 3' |
| S^x4^ fw  S^x4^ rev | 5' GGAATTCCTCGACGGTATCGATAA 3'  5' GCTTATCGGGGCTGCAGGAATT 3' |
| Ras fw  Ras rev | 5' GAGGGATTCCTGCTCGTCTTCG 3'  5' GTCGCACTTGTTACCCACCATC 3' |
