# Supplementary figures and images for: Role of Su(Hw) zinc finger 10 and interaction with CP190 and Mod(mdg4) proteins in recruiting the Su(Hw) complex to chromatin sites in Drosophila
Source: PLoS One. 2018 Feb 23;13(2):e0193497. doi: 10.1371/journal.pone.0193497 (PMC5825117; doi:10.1371/journal.pone.0193497)

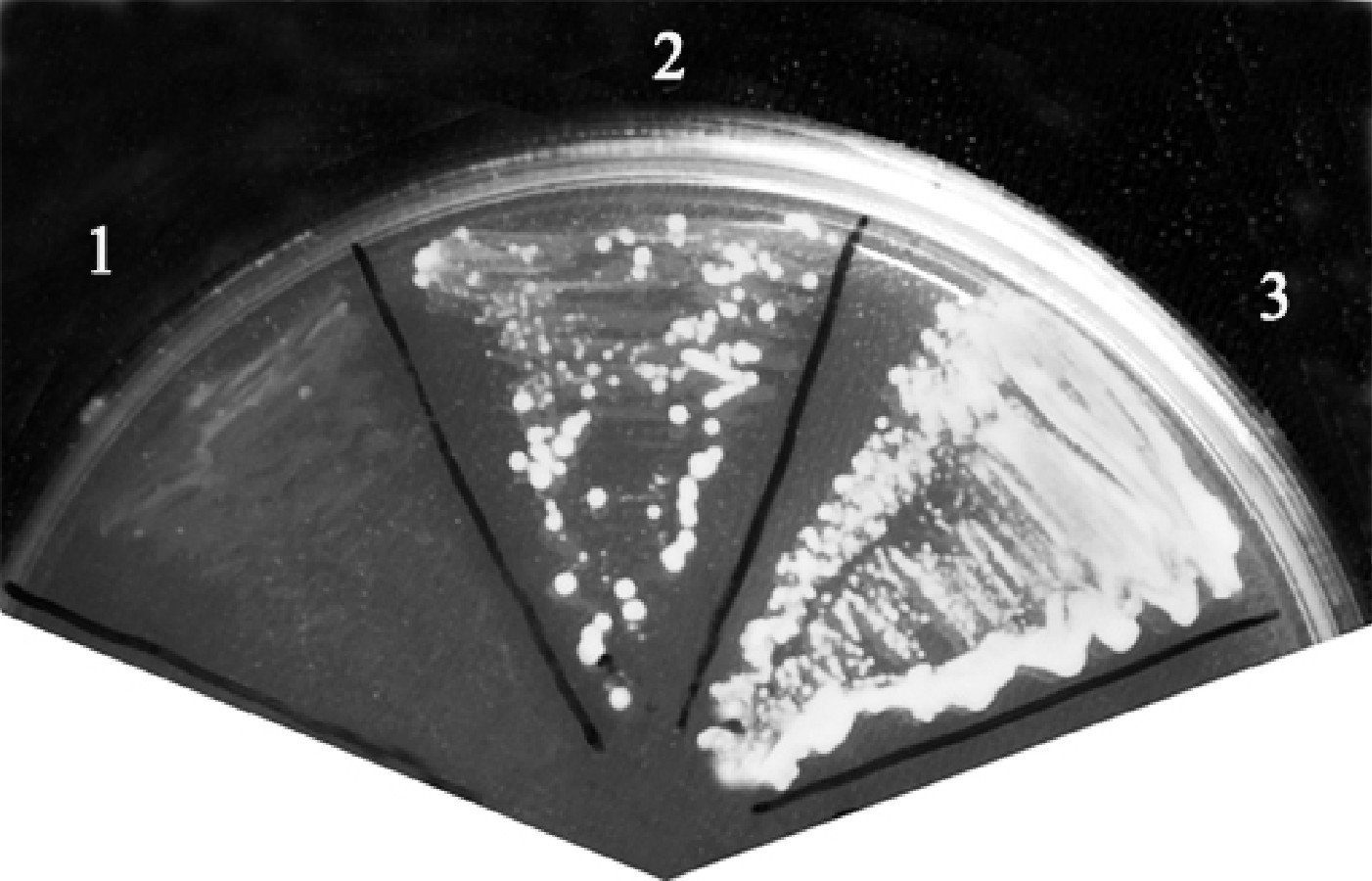

Supplement: S1 Fig — (1)—no interaction « − », (2)—« + », (3)—« ++ ». (TIF) [file pone.0193497.s003.tif]

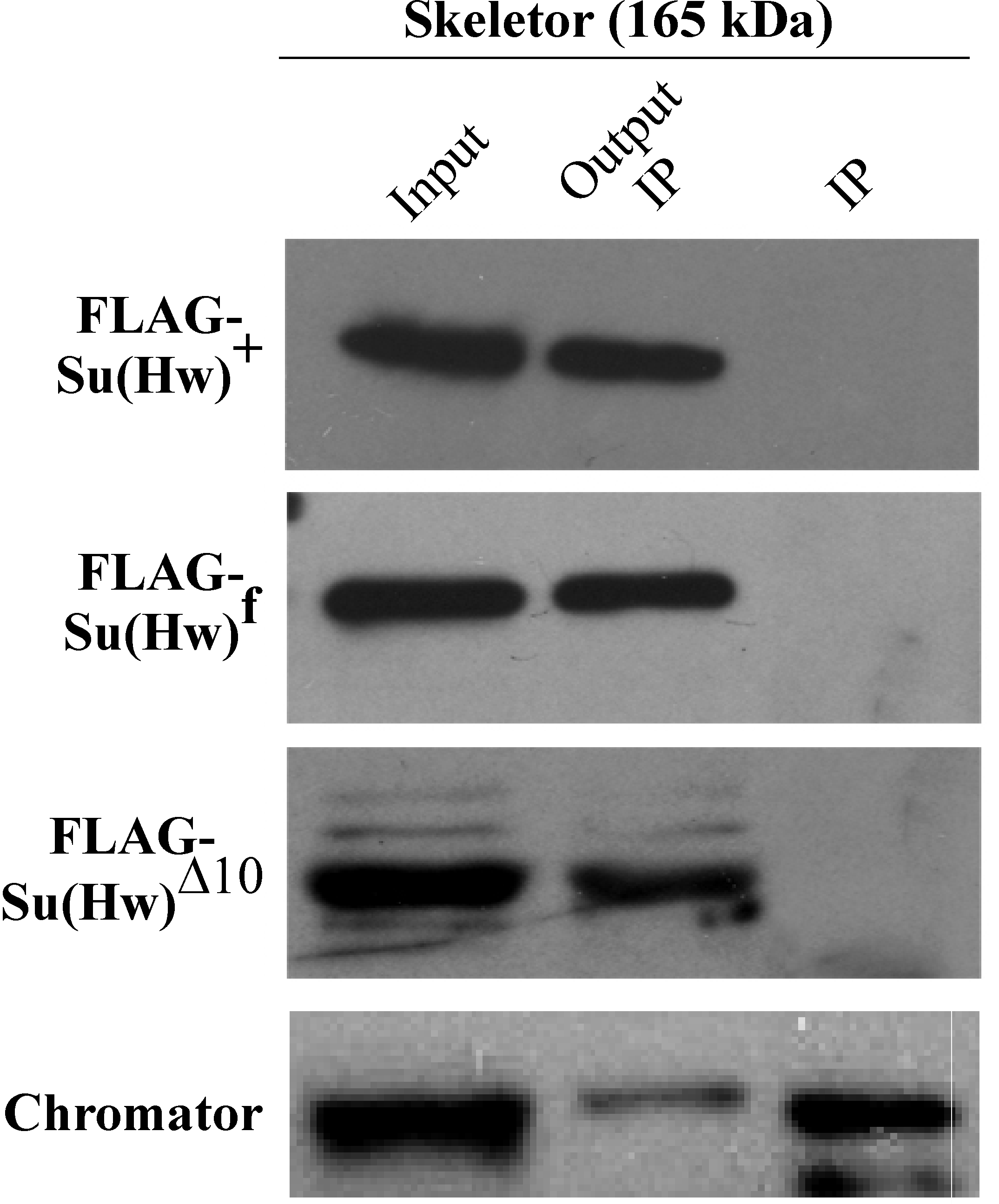

Supplement: S2 Fig — The FLAG-Su(Hw)+, FLAG-Su(Hw)f, and FLAG-Su(Hw)Δ10 were expressed in the S2 cells. The immunoprecipitated complexes were washed with buffers containing 150 mM NaCl before loading onto the SDS-PAGE for Western blot analysis. The PVDF membrane was probed with antibodies against the Skeletor protein. Each panel represents a single FLAG immunoprecipitation experiment with the particular Su(Hw) variant. Su(Hw) variants do not interact with the Skeletor protein. The bottom panel shows the result of immunoprecipitation between Chromator and Skeletor proteins that interact with each other [83]. All results were reproduced in two independent experiments. (TIF) [file pone.0193497.s004.tif]

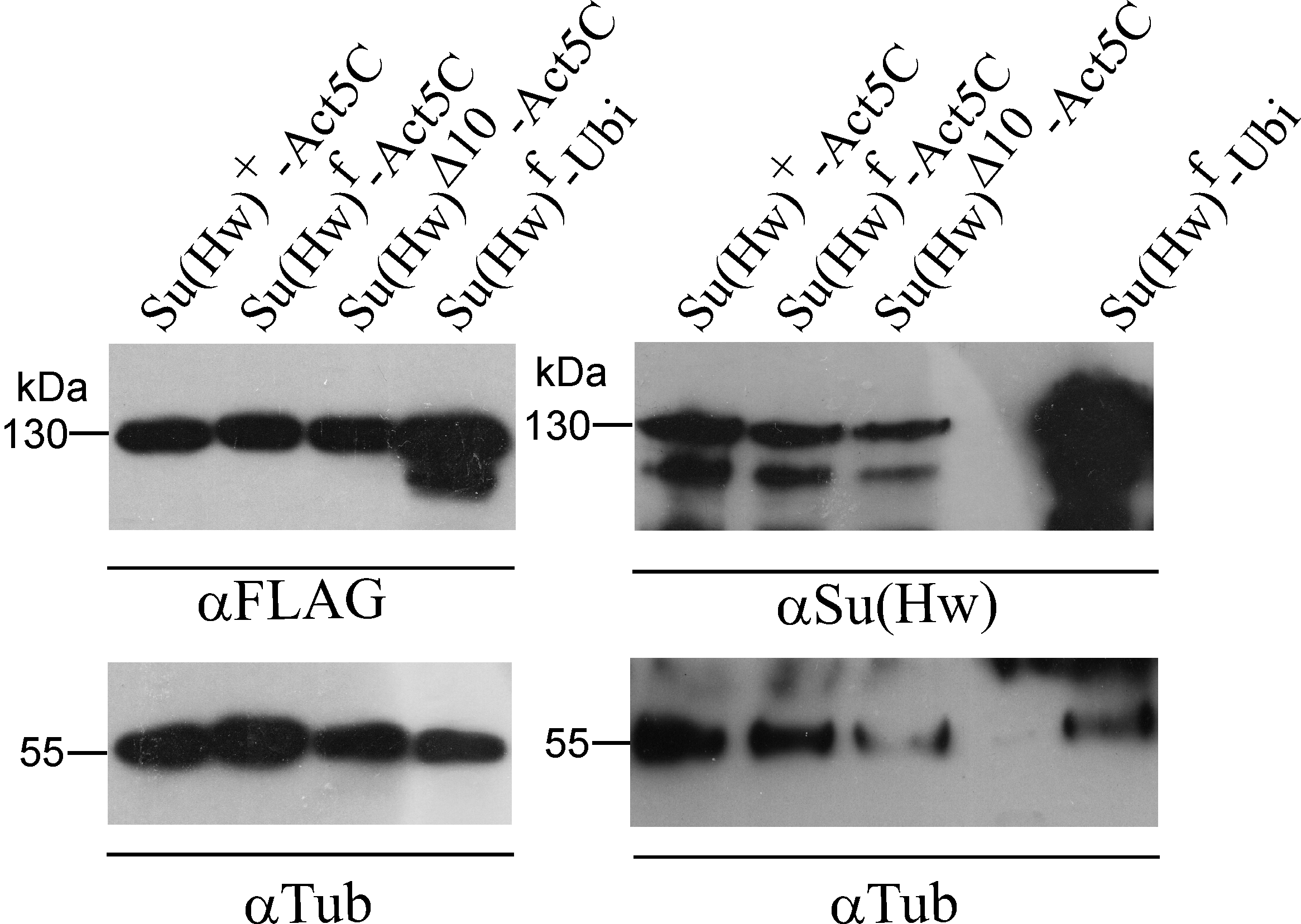

Supplement: S3 Fig — Su(Hw) isolated from adult flies as described (Gdula et al., 1997) [84] was resolved by electrophoresis in 7.5% SDS-PAAG, electroblotted onto a PVDF membrane, and probed with antibodies against Su(Hw) or FLAG epitope (designated αSu(Hw) and αFLAG, respectively). Anti-tubulin staining (αTub) was used as loading control. The name of alleles included in analysis are indicated above the figure. Expression analysis of transgenic lines was performed on the y2scD1ct6; su(Hw)v/ su(Hw)e04061 background. (TIF) [file pone.0193497.s005.tif]

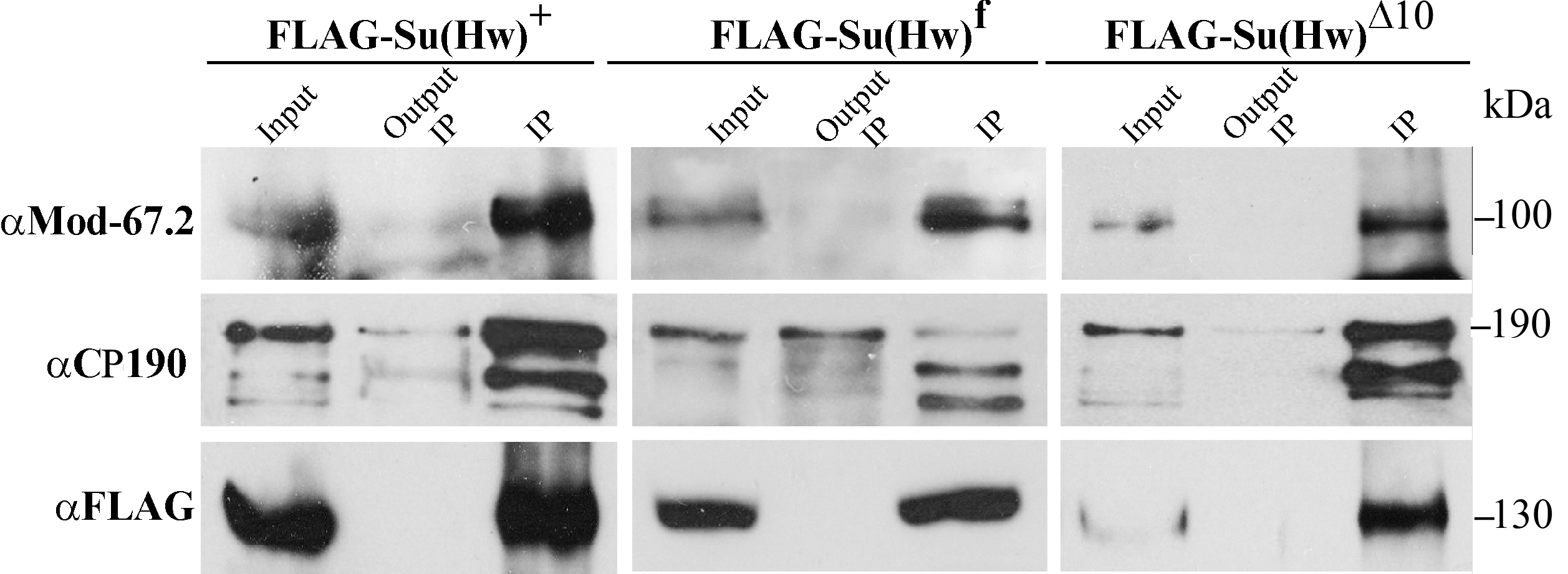

Supplement: S4 Fig — The immunoprecipitated complexes were washed with 150 mM NaCl-containing buffers before loading onto SDS-PAGE for Western blot analysis. The PVDF membrane was consecutively probed with antibodies against the indicated proteins (CP190 or Mod-67.2) or FLAG epitope. "Input" is the input fraction (10% of lysate used for immunoprecipitation); "Output IP," the supernatant after immunoprecipitation; "IP," the immunoprecipitate. The names of alleles included in analysis are indicated above the figure. Analysis of transgenic lines was performed on the y2scD1ct6; su(Hw)v/ su(Hw)e04061 background. All results were reproduced in three independent experiments. (TIF) [file pone.0193497.s006.tif]

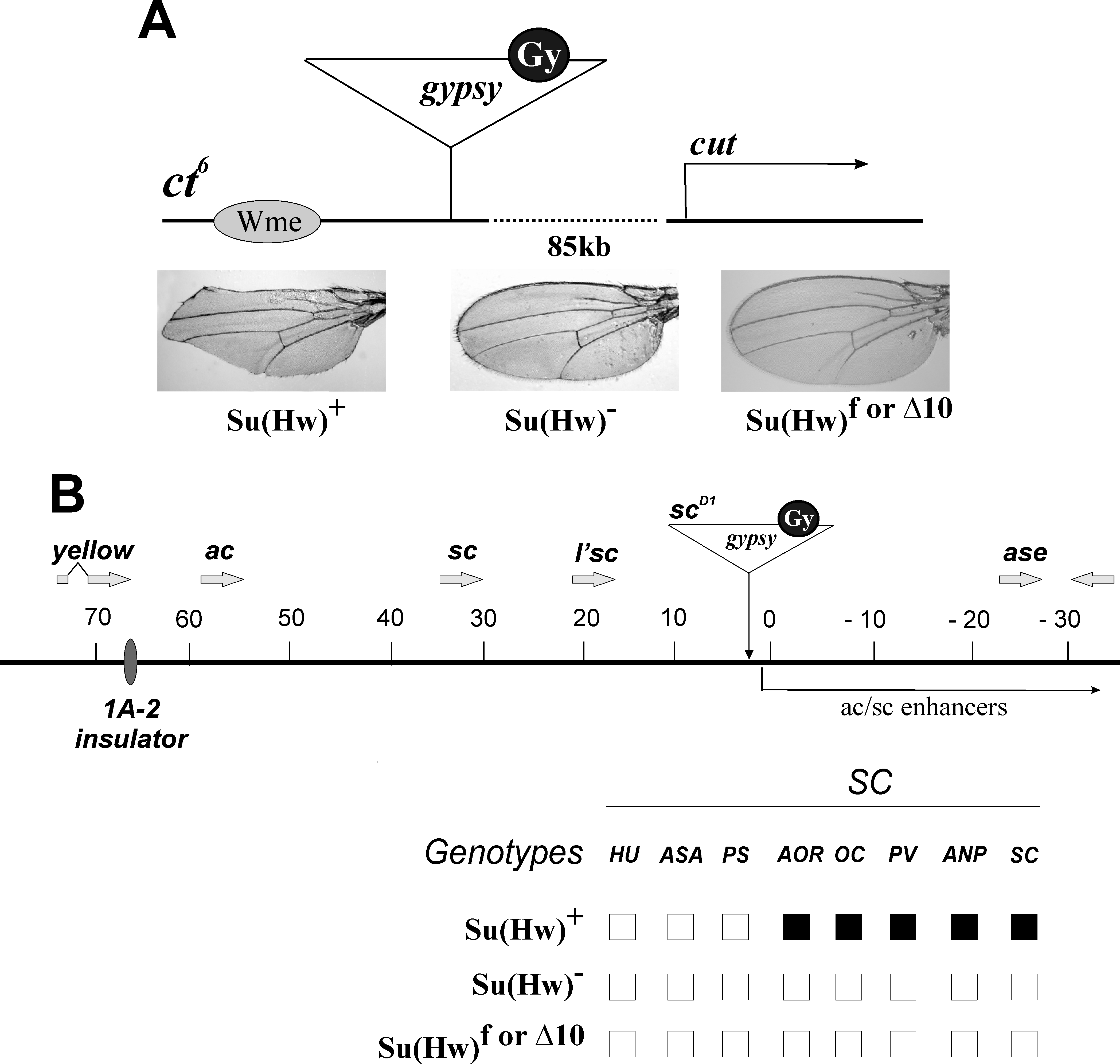

Supplement: S5 Fig — (A) Schemes (not to scale) of the ct6 allele. The gray oval (Wme) indicates the wing margin enhancer controlling cut expression in the wings. The transcription start site indicated by arrowheads. The gypsy insertion is shown as a triangle with the black circle (Gy) marking Su(Hw) binding sites. The names of alleles included in analysis are under the photos showing the cut wing phenotype. (B) Scheme of the yellow/ac/sc region in scD allele. The coordinates of the AS-C region are as defined by Campuzano et al. (1985) [76]. The gypsy (scD1) insertion is shown as a triangle with an arrow. Thick horizontal gray arrows show the positions and direction of yellow and AS-C gene transcripts. Filled oval indicate the endogenous Su(Hw) insulator. The standard nomenclature for each bristle is as follows: HU, humeral; AOR, anterior orbital; PS, presutural; ASA, anterior supra-alar; OC, ocellar; PV, postvertical; ANP, anterior notopleural; SC, scutellar. Only the bristles affected in the ac and sc mutations are shown. Empty boxes indicate that the corresponding bristles are present (wild-type phenotype). Filled boxes indicate the absence of the corresponding bristle(s) in more 90% of the flies. (TIF) [file pone.0193497.s007.tif]

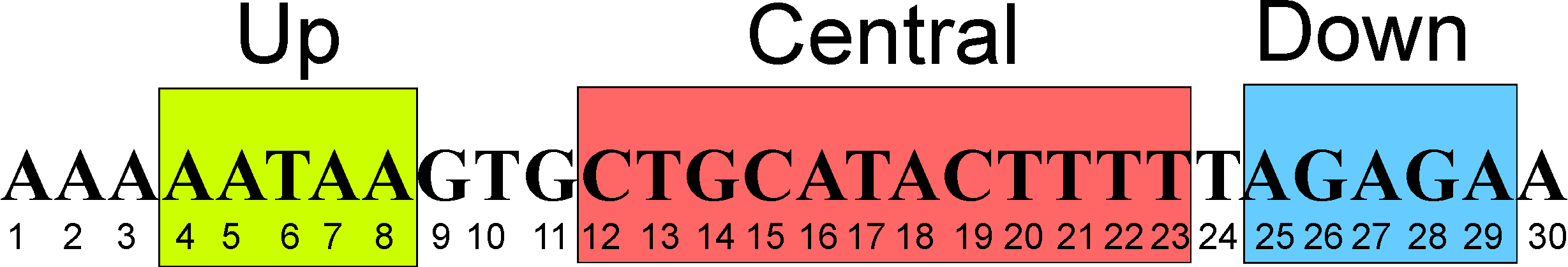

Supplement: S6 Fig — This oligonucleotide contains gypsy insulator sequences corresponding to binding site 3 and includes the consensus sequence with three modules that represents the highest-affinity Su(Hw) binding site [57]. (TIF) [file pone.0193497.s008.tif]

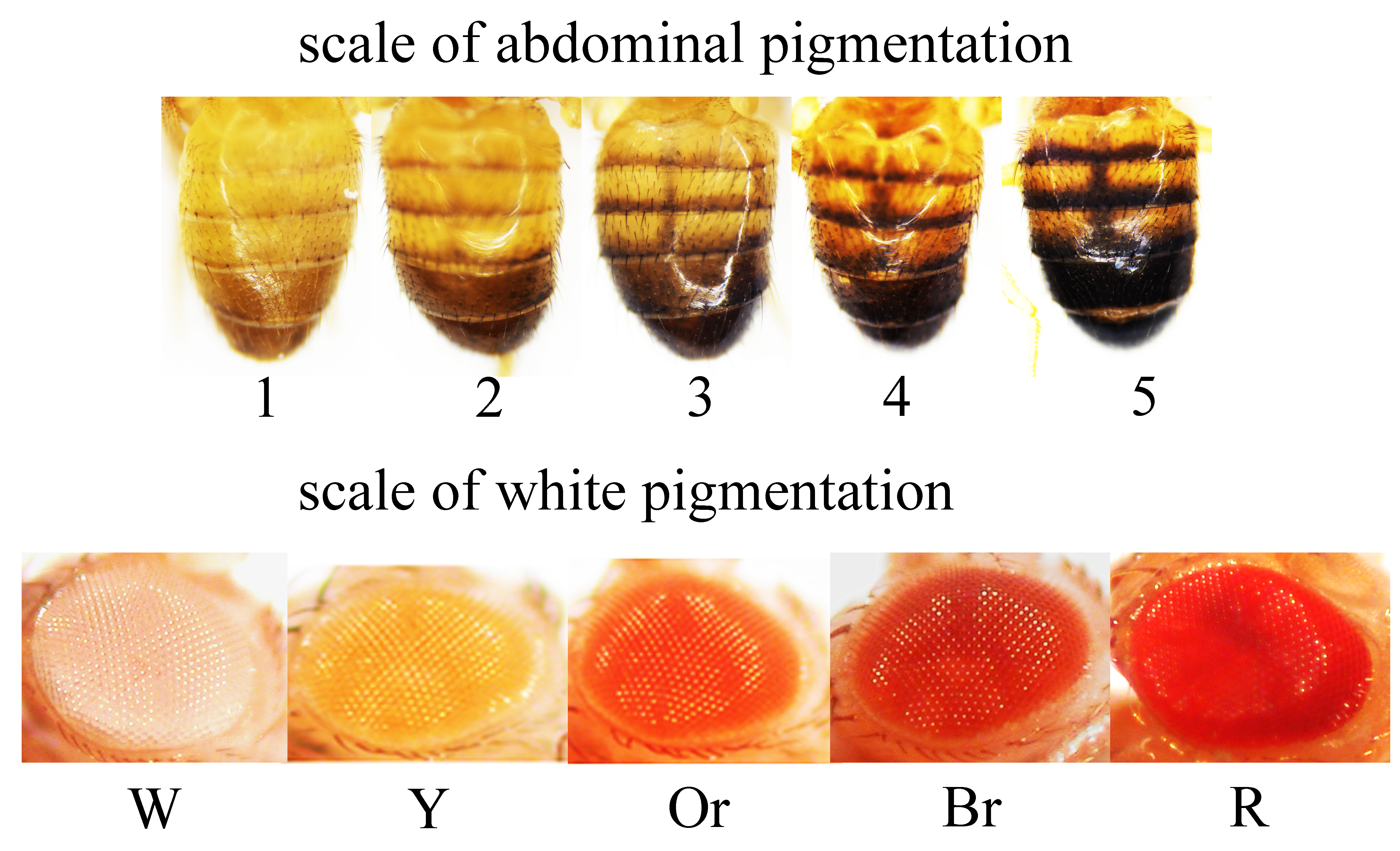

Supplement: S7 Fig — Photos represent the abdominal and eyes pigmentation in the 3-day-old males. Numbers indicate the scores of yellow expression in the abdominal segments, which ranged from 1 (pigmentation as in y1 allele) to 5 (pigmentation as in wild-type flies). Wild-type white expression determined the bright red eye color (R); in the absence of white expression, the eyes were white (W). Intermediate levels of pigmentation are yellow (Y), orange (Or), and brownish red (Br). (TIF) [file pone.0193497.s009.tif]
